# Gene Ontology Enrichment Improves Performances of Functional Similarity of Genes

**DOI:** 10.1038/s41598-018-30455-0

**Published:** 2018-08-14

**Authors:** Wenting Liu, Jianjun Liu, Jagath C. Rajapakse

**Affiliations:** 1 Human Genetics, Genome Institute of Singapore, Singapore, Singapore; 2 School of Computer Science and Engineering, Nanyang Technological University, Singapore, Singapore

## Abstract

There exists a plethora of measures to evaluate functional similarity (*FS*) between genes, which is a widely used in many bioinformatics applications including detecting molecular pathways, identifying co-expressed genes, predicting protein-protein interactions, and prioritization of disease genes. Measures of *FS* between genes are mostly derived from Information Contents (IC) of Gene Ontology (GO) terms annotating the genes. However, existing measures evaluating IC of terms based either on the representations of terms in the annotating corpus or on the knowledge embedded in the GO hierarchy do not consider the enrichment of GO terms by the querying pair of genes. The enrichment of a GO term by a pair of gene is dependent on whether the term is annotated by one gene (i.e., partial annotation) or by both genes (i.e. complete annotation) in the pair. In this paper, we propose a method that incorporate enrichment of GO terms by a gene pair in computing their *FS* and show that GO enrichment improves the performances of 46 existing *FS* measures in the prediction of sequence homologies, gene expression correlations, protein-protein interactions, and disease associated genes.

## Introduction

Gene ontology (GO) provides a controlled vocabulary of gene functions and molecular attributes that are arranged in a directed acyclic graph (DAG) representing semantic relations among GO terms. GO is often used to interpret results and make inferences of biological experiments. Functional similarity (*FS*) between two genes is inferred from GO terms annotating the genes and is widely adopted in detecting and interpreting genetic interactions, functional interactions, protein-protein interactions^1^, biological pathways^2,3^, prioritization of disease genes^4^, and disease similarities^5^. Most *FS* measures in the literature are computed using information contents (IC) of GO terms annotating the querying pair of genes. The IC of a GO term is evaluated using either the representation of GO terms in the annotation corpus associated with a species (corpus-based methods) or the structure of the DAG (structure-based methods).

Functional similarity between two genes is given by the common information or semantic similarity of the GO terms annotating the two genes. Semantic similarity (SS) among GO terms is generally evaluated by the ICs of common ancestor terms as ancestors subsume semantic concepts of descendants due to the hierarchical nature of the DAG. Corpus-based SS measures such as Resnik^6^, Lin^7^, Nunivers^8^, and Schlicker^9^ are based on the ICs of the most informative common ancestor, and XGraSM^10^ and TopoICSim^11^ have extended Lin and Nunivers measures to include IC of all the common ancestors. Structure-based methods such SORA^12^ and WIS^13^ determine the ICs of a GO term based on the number of descendants and/or the depth of the term in the DAG. Semantic similarity of a GO term set is derived from the ICs of (i) individual terms, (ii) pairs of terms, or (iii) the term set. The ICs of a term set is derived combining individual or pair of terms by using statistical averaging measures or Tversky’s ratios^14^.

**Figure 1:**
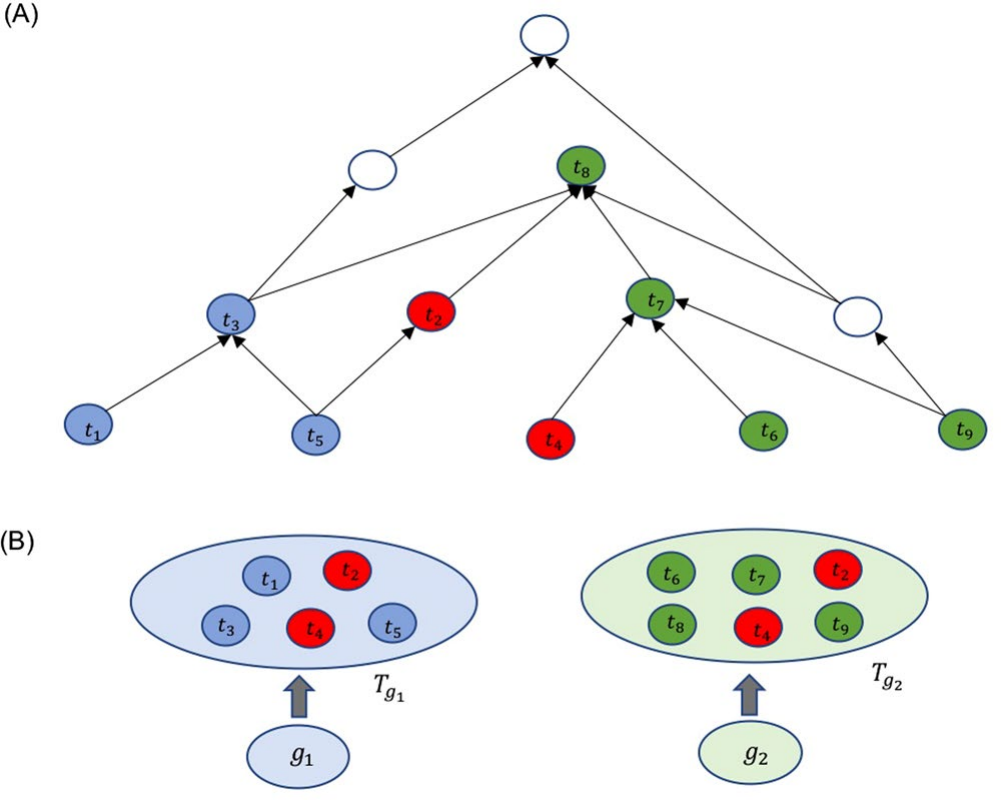
Illustration of a DAG representing GO terms annotating two genes. (**A**) The DAG representing GO terms that annotates the two genes, and (**B**) the two genes *g*_1_ and *g*_2_ and their GO term sets ${T}_{{g}_{1}}$ and ${T}_{{g}_{2}}$. The *FS* is derived as the semantic similarity or the common information contents (IC) in the two term sets. Our approach takes care of differential enrichment of GO terms by the querying gene pair: for example, blue terms annotates only *g*_1_, green terms annotates only gene *g*_2_, and red terms annotates both genes *g*_1_ and *g*_2_.

Functional similarity of two genes are measured by ICs of common GO terms annotating the genes, which are evaluated purely based on the annotations of the corpus or the expert knowledge embedded in the DAG. Figure 1 illustrates how two genes are represented in a DAG by their GO terms and as seen, some GO terms in the DAG annotate only one gene while others annotate both genes or none. However, existing IC measures ignore the local context or how GO terms are represented in the querying gene pair. For example, when measuring *FS* of a gene pair, GO terms annotating both genes are more likely to be enriched than those annotating only one gene. To overcome this drawback of existing *FS* measures, we propose to incorporate GO-enrichment by querying gene pair in the computation of IC of a GO term. Specifically, in the context of two genes, the probability of a GO term is defined as the joint probability of the term as inferred by background corpus and as annotated by two querying genes. We investigate the effect of introducing GO enrichment on 46 existing *FS* measures and demonstrate that enriched *FS* (FS*) measures outperform the prediction of sequence homologies, gene expression correlations, protein-protein interactions, and disease-associated genes in majority of the cases.

## Results

### Performance on FS* on all datasets

We assessed performances of FS* measures on benchmark datasets for predicting sequence similarities, gene expression (GE) correlations, protein-protein interactions (PPI), disease genes (DG) and compared with those of corresponding *FS* measures. Table 1 shows one-sided *p*-values of the improvement of performances of all the experiments on five benchmark datasets, by using Wilcoxon signed rank tests^15^. As seen, FS* measures showed significant improvement over *FS* measures in the prediction of disease genes on 138 experiments, protein interactions on 138 experiments of yeast PPI data and 138 experiments of human PPI data, gene co-expressions on 138 experiments of yeast GE data, and sequence similarities on 276 experiments on CESSM dataset; and on all 828 experiments. Irrespective of the ontology (BP, MF, or CC) and the type of *FS* measure, incorporation of GO enrichment significantly improved the prediction of sequence similarities, gene co-expression patterns, protein-protein interactions, and disease associated genes.

**Table 1:** Details of five datasets and statistical significances of the improvement of performances by FS^*^ over corresponding *FS* measures on predicting disease genes, protein interactions on yeast PPI dataset and yeast GE dataset, gene co-expressions on yeast GE dataset in different ontological domains (BP, MF, and CC), and sequence similarities on CESSM datasets (ECC, Pfam, and SeqSim).

Data Type	Data Sets	^#^Protein pairs	ontology	^#^Experiments	*p*-value
Disease Genes	DG_BP; DG_MF; DG_CC	6084	BP; MF; CC	138	5.619e-08
Yeast PPI	PPI_BP; PPI_MF; PPI_CC	8654; 7166; 8852	BP; MF; CC	138	2.885e-07
Human PPI	PPI_BP; PPI_MF; PPI_CC	2408; 2576; 2108	BP; MF; CC	138	3.528e-03
Yeast GE	GE_BP; GE_MF; GE_CC	4800	BP; MF; CC	138	6.912e-09
CESSM	ECC; Pfam; SeqSim	13430	BP; MF	276	4.94e-16
Total	DG; PPI; GE; ECC; Pfam; SeqSim	35376; 34056; 35274	BP; MF; CC	828	<2.2e-16

### Performance of FS* measures on individual datasets

Table 2 lists 46 FS measures that are computed based on ICs of individual terms, pairs of terms, and the set of terms annotating the genes. Lin^7^ (L), Nunivers^8^ (N), Schlicker^9^ (C), and extended (XGraSM^10^) Lin (XL) and Nunivers (XN), Zhang^16^ (Z) GO-universal^8^ (U) and Wang^17^ (W) are corpus-based methods using IC son individual terms or pairs of terms (i.e., SS). Functional similarities of the gene pair are computed by combining ICs of annotating GO terms by using GIC^1^ (Jaccard index), DIC^8^ (dice index), and UIC^8^ (universal index) on individual terms, and Average (AVG), Maximum (MAX), Best-Match Average (BMA) and Average Best-Matches (ABM) on pairs of terms; SORA^12^ (R) and WIS^13^ (I) are structure-based methods that define ICs for individual terms from information from DAN and compute ICs on the whole term set by using Overlap Ratio (OR) and Intersection to Union Ratio (IUR).

**Table 2:** Details of 46 FS measures. The types of IC/SS and methods used to compute *FS* measures: GIC^1^ (Jaccard index), DIC^8^ (dice index), and UIC^8^ (universal index) for individual terns; Average (AVG), Maximum (MAX), Best-Match Average (BMA) and Average Best-Matches (ABM) for measures based on pairs of terms; and Overlap Ratio (OR) and Intersection to Union Ratio (IUR) for measures based on sets of terms.

Acronyms	IC/SS	*FS* measures
U	GO-universal^8^	U_ABM_, U_BMA_, U_MAX_, U_AVG_, U_DIC_, U_GIC_, U_UIC_
Z	Zhang^16^	Z_ABM_, Z_BMA_, Z_MAX_, Z_AVG_, Z_DIC_, Z_GIC_, Z_UIC_
W	Wang^17^	W_ABM_, W_BMA_, W_MAX_, W_AVG_, W_DIC_, W_GIC_, W_UIC_
N	Nunivers^8^	N_ABM_, N_BMA_, N_MAX_, N_AVG_
XN	Extended Nunivers^10^	XN_ABM_, XN_BMA_, XN_MAX_, XN_AVG_
L	Lin^7^	L_ABM_, L_BMA_, L_MAX_, L_AVG_
XL	Extended Lin^10^	XL_ABM_, XL_BMA_, XL_MAX_, XL_AVG_
S	Schlicker^9^	S_ABM_, S_BMA_, S_MAX_, S_AVG_
D	Direct-term based^18^	D_DIC_, D_GIC_, D_UIC_
R	SORA^12^	R_OR_
I	WIS^13^	I_IUR_

Tables 3, 4, 5 and 6 lists the top five performers of *FS*/FS* measures on each dataset. As seen from the tables, the best performers (ranked by AUC values of prediction) are mostly FS* measures: (i) I_IUR_* on predicting sequence similarity on both BP and MF ontology, Pfam similarity on MF ontology, and PPIs of yeast on CC ontology; (ii) R_OR_* on predicting ECC similarity on MF ontology, gene co-expressions of yeast on both BP and MF ontology, and disease genes on both BP and MF ontology; (iii) W_ABM_* on predicting Pfam similarity on BP ontology and PPIs of human on CC ontology; Z_BMA_* on predicting PPIs of human on BP ontology; and (iv) XN_BMA_* on predicting ECC similarity on BP ontology, PPIs of yeast on BP ontology, and disease genes on BP ontology. For predicting PPIs of yeast on MF ontology, both D_UIC_ and D_UIC_* performed best with quite similar AUC score of 0.6930 and 0.6928, respectively, followed by D_DIC_* and D_GIC_* with AUC score both of 0.6926. S_ABM_ performed best on predicting human PPIs from MF ontology.

**Table 3:** Top five performers of *FS* and FS* measures on predicting ECC, Pfam, and SeqSim similarities of protein pairs of CESSM datasets, using BP and MF ontologies.

Datasets	Methods	Correlation	Datasets	Methods	Correlation	Datasets	Methods	Correlation
ECC_BP	**XN**_BMA_^*^	**0.4748**	Pfam_BP	**W**_ABM_^*^	**0.5261**	SeqSim_BP	**I**_IUR_^*^	**0.8028**
	**XN**_**BMA**_	**0.4748**		W_BMA_^*^	0.5223		I_IUR_	0.7927
	XL_BMA_^*^	0.4748		R_OR_^*^	0.5199		R_OR_	0.7884
	XL_BMA_	0.4708		I_IUR_^*^	0.5005		W_ABM_^*^	0.7741
	N_BMA_^*^	0.4651		R_OR_	0.4933		R_OR_^*^	0.7738
	**R**_OR_^*^	**0.7828**		**I**_IUR_^*^	**0.6961**		**I**_IUR_^*^	**0.7217**
	W_BMA_^*^	0.7665		R_OR_^*^	0.6829		I_IUR_	0.7165
ECC_MF	XN_BMA_^*^	0.7567	Pfam_MF	I_IUR_	0.6627	SeqSim_MF	R_OR_	0.6505
	XN_BMA_	0.7525		R_OR_	0.6565		D_GIC_^*^	0.6358
	N_BMA_^*^	0.7525		W_ABM_^*^	0.6283		D_GIC_	0.6285

**Table 4:** Top five performers of *FS* and FS* measures predicting protein-protein interactions of human and yeast PPI datasets, using three ontologies: BP, MF, and CC.

Datasets	Methods	AUC	Datasets	Methods	AUC	Datasets	Methods	AUC
human PPI_BP	**Z**_BMA_^*^	**0.8750**	human PPI_MF	S_ABM_	0.7787	human PPI_CC	**W**_ABM_^*^	**0.7775**
	Z_BMA_	0.8747		L_ABM_	0.7777		W_BMA_^*^	0.7697
	N_BMA_^*^	0.8739		S_ABM_^*^	0.7771		I_IUR_^*^	0.7678
	N_BMA_	0.8737		L_ABM_^*^	0.7762		U_ABM_^*^	0.7658
	S_BMA_^*^	0.8721		N_ABM_	0.7718		U_ABM_	0.7657
yeast PPI_BP	**XN**_BMA_^*^	**0.8565**	yeast PPI_MF	D_UIC_	0.6930	yeast PPI_CC	**I**_IUR_^*^	**0.8248**
	XN_MAX_^*^	0.8563		D_UIC_^*^	0.6928		I_IUR_	0.8158
	XL_MAX_^*^	0.8561		D_DIC_^*^	0.6926		R_OR_^*^	0.8143
	XN_MAX_	0.8559		D_GIC_^*^	0.6926		U_ABM_^*^	0.8072
	XN_BMA_	0.8559		D_GIC_	0.6916		N_ABM_^*^	0.8068

**Table 5:** Top five performers of *FS* and FS^*^ measures predicting gene co-expressions on yeast GE dataset, using three ontologies: BP, MF, and CC.

Datasets	Methods	Correlation	Datasets	Methods	Correlation	Datasets	Methods	Correlation
yeast GE_BP	**R**_OR_^*^	**0.2927**	yeast GE_MF	**R**_OR_^*^	**0.2138**	yeast GE_CC	**Z**_DIC_^*^	**0.4263**
	**D**_GIC_^*^	**0.2877**		R_OR_	0.2087		Z_DIC_	0.4253
	R_OR_	0.2876		D_GIC_^*^	0.2023		Z_GIC_^*^	0.4236
	Z_GIC_^*^	0.2875		D_GIC_	0.2022		Z_GIC_	0.4233
	D_GIC_	0.2873		D_DIC_^*^	0.2008		Z_UIC_	0.4229

**Table 6:** Top five performers of *FS* and FS^*^ measures predicting disease genes on benchmark dataset, using three ontologies: BP, MF, and CC.

Datasets	Methods	AUC	Datasets	Methods	AUC	Datasets	Methods	AUC
Disease Genes_BP	**XN**_BMA_^*^	**0.8065**	Disease Genes_MF	**R**_OR_^*^	**0.7541**	Disease Genes_CC	**R**_OR_^*^	**0.7064**
	R_OR_	0.8062		U_BMA_^*^	0.7357		U_BMA_	0.7032
	XN_BMA_	0.8058		U_BMA_	0.7357		R_OR_	0.7031
	I_IUR_	0.8030		N_BMA_^*^	0.7344		U_BMA_^*^	0.7029
	N_BMA_^*^	0.8019		N_BMA_	0.7330		W_BMA_	0.7006

We notice that the best performers are mostly FS* measures derived from structure-based IC measures (I_IUR_* and R_OR_*), corpus-based IC measures that considered ancestors of the terms (W_ABM_* and Z_BMA_*) or extended corpus-based measures (XN_BMA_*). This indicates that using only the information of annotating corpus is insufficient and both the structure of DAG and GO enrichment by the querying gene pair are essential for determining *FS* between genes.

Supplementary Tables 1–6 give the details of performances of FS* over all 46 FS measures in predicting sequence similarities, gene co-expressions, protein-protein interactions and disease genes. The tables list correlations or AUC scores of the measures, percentages improvement of FS* over *FS*, and statistical significances of improvement on every dataset. The significant improvements at FDR < 0.01 are indicated in bold and the significant drops in performance are marked in red; for each dataset, top *FS* and top FS* performers are indicated in bold.

Our results show that GO enrichment improves on almost all 46 *FS* measures on all the datasets, except inferring human PPIs on MF ontology. The corpus-based measures (Lin^7^, Nunivers^8^, GO-universal^8^, Wang^17^, Zhang^16^) and graph-based extensions of corpus-based measures (XGraSM^10^ of Lin^7^ and Nunivers^8^) were improved significantly with GO enrichment on most datasets. In general, BMA and ABM methods provided best performances and performed equally well on most semantic similarity measures. Adaptation of efficient correction factors improved the performance on some measures: Schlicker^9^ uses the IC value of MICA and does not significantly improve the performance of the Lin^7^ approach; XGraSM^10^ uses all common informative ancestors to correct Lin^7^ and Nunivers^8^ approaches in order to improve their performances. Thus, including common informative ancestors in the conception of SS improves performance, especially for approaches including only the features of child terms in the computation of IC such as Zhang^16^, Wang^17^, SORA^12^ and WIS^13^ measures.

As FS* measures differently treats GO terms uniquely annotating (i.e., annotating one gene) and GO terms commonly annotating (i.e., annotating both genes) the querying genes, measures including both types of terms are significantly improved with GO enrichment: for example, Lin^7^, Nunivers^8^, and Direct-term based^18^ measures consider both common terms and individual terms; GO-universal^8^ measure considers all children terms (common or individual terms); and Zhang^16^, Wang^17^, SORA^12^ and WIS^13^ measure consider all ancestors (common terms) and children terms (common or individual terms). Especially, Wang^17^ measures (W_ABM_, W_BMA_) improved significantly on capturing sequence homology with a correlation improvement of 8% of ECC, 25% of Pfam, 34% of SeqSim on MF ontology; and 13% of Pfam, 16% of SeqSim on BP ontology while Wang^17^ measures (W_ABM_, W_BMA_, W_MAX_) improved significantly for GE correlations on BP and MF with a correlation improvement of 3.5% on BP, and 16% on MF. Wang^17^ measure (W_AVG_) also improved most significantly for inferring human PPIs on CC with 3% AUC improvement, yeast PPIs on BP and CC with AUC improvement 4% and 3%, respectively. SORA^12^ approach (R_OR_) improved most significantly for predicting yeast PPIs on MF ontology with AUC improvement 2%, disease genes on MF ontology with AUC improvement 4%. WIS^13^ approach (I_IUR_) improved most significantly for GE correlations on BP, MF, and CC with correlation improvement of 5%, 6%, and 5%, respectively. GO-universal approach (U_AVG_) improved most significantly (labelled as green) for GE correlations on BP and CC with correlation improvement of 3% and 10%, respectively; and inferred human PPIs on BP, yeast PPIs on CC with AUC improvement both 1%.

Direct term-based D_GIC_^18^ and D_DIC_^18^ are improved most significantly for inferring human PPIs on BP and MF, yeast PPIs on BP and CC, GE correlations on BP and CC, disease genes on CC. Out of all 46 FS measures, the performance of measure related to UIC measure didn’t improve with GO enriched FS* measures. This is because the UIC measure does not discriminate common terms and unique terms while the enrichment is manifested by the differences between common and unique terms. UIC is defined as the sum of IC of the terms annotated by both genes, divided by the maximum of the sum of ICs annotated individual genes and GIC is defined as the sum of ICs of GO terms annotated by both genes, divided by the sum of ICs of terms annotated by individual genes. The enriched IC term leads to increase the ICs of the terms that are annotated by both genes more than those annotated by one gene. When there are only a few terms annotated by both genes, GIC*/UIC* do not perform as good as UIC. Therefore, the *FS* with GIC*/UIC* lead to quality loss when predicting sequence similarity as seen in Supplementary Table 2. The loss in predicting with GIC* is smaller than the loss in predicting with UIC*.

Supplementary Figs 1–3 show ROC curves of overall best performers (I_IUR_, R_OR_, XN_BMA_, Z_BMA_ and D_GIC_) of *FS* to FS*, predicting human and yeast PPIs, disease genes on three ontologies, respectively. As seen, most of top performers are FS*, underscoring that GO enrichment indeed improves performance of best *FS* performers on all the datasets.

## Conclusions

Many *FS* measures have been proposed using GO annotation to quantify similarities between genes for exploitation and validation of biological knowledge embedded in omics data. These measures were derived based on the topological structure of GO semantics and/or the GO annotations of the genes/proteins annotating (background) corpus. However, the representativeness of GO terms in the two querying genes has not been considered in evaluating *FS* measures. In other words, differential enrichment of the terms annotating one gene and the terms annotating two genes in the querying pair has been ignored in the existing *FS* measures. We proposed an enriched *FS* measure, FS*, that can be used to incorporates the enrichment of GO terms by querying genes in the existing *FS* measures and demonstrated improved performances of FS* measures in the prediction of sequence similarities, protein-protein interactions, gene co-expressions, and disease associated genes.

We tested GO enrichment on 46 *FS* measures on five benchmark datasets including sequence similarities of the CESSM dataset, yeast GE data, human and yeast PPI data, and disease genes, and presented comparison of performances of *FS* and FS* measures. Results indicate that FS* outperforms *FS* measures in a vast majority of the experiments. We conclude that consideration of GO enrichment by the querying genes is an essential step for computation of *FS* between genes. As seen, *FS* measures including both commonly and individually annotating terms, the performances of FS* between genes improved much significantly over *FS* measures. We also noticed that FS* significantly outperformed on datasets containing a lot of uniquely annotated genes (i.e., those annotated by the terms in the low levels of GO hierarchy).

Enriched FS* of structure-based measures (I_IUR_ and R_OR_), corpus-based methods using DAG structure (W_ABM_ and Z_BMA_), or graph-based extensions of corpus-based measures (XN_BMA_) achieved best performances on all the benchmark datasets except predicting human and yeast PPIs on MF ontology. This indicate that introducing the annotations of the corpus and the querying pair of genes in the structure-based IC measures gives accurate *FS* measures, underscoring the need for incorporating the representativeness of querying genes.

Enriched FS* is easily adapted to and generally improves the performance of any *FS* measure that uses ICs of GO terms. On the other hand, the accuracy of GO annotation naturally limits the performance of existing *FS* measures as they do not consider both the local context of two genes and the background distribution of terms in the annotating corpus. Our experiments suggest that the local context of querying genes is sensitive to the missing and spurious terms in the GO annotating corpus. One could extend our method to evaluate the functional coherence of gene sets, which will have applications in the detection of functional modules or pathways. FS* measures more accurately identify functionally similar genes than *FS* measures and will provide more reliable computational evidences for finding new pathways and disease genes. We conclude that the GO enrichment is an essential step when assessing *FS* of two genes and a set of genes.

## Methods

### Data Sets

Molecules with sequence similarities show similar functions or MF ontology, and with similar gene expressions are likely to belong to same pathway or so have similar BP ontology. Interacting proteins are located in the same cellular location and so have the similar CC ontology. Therefore, we evaluated the performances of FS* measures by their correlations with sequence similarities, gene co-expressions, protein-protein interactions, and disease association of genes on benchmark datasets.

### Correlation with sequence similarity

Various studies have shown that molecules with sequence similarities have similar ontological annotations^19^, so we used sequence similarities to demonstrate the goodness of *FS* measures^20,21^. For BP and MF, we downloaded sequence similarities of selected human proteins with known relationships from CESSM^22^ online tool (http://xldb.di.fc.ul.pt/tools/cessm/) and compared the performances of different *FS* measures predicting sequence similarities. The CESSM website provides a list of protein pairs and similarities between pairs of proteins, using three distinct evaluations: sequence similarity (SeqSim), Pfam domain similarity, and enzyme commission class (ECC) similarity. The goodness of prediction was evaluated by the correlations between protein similarities captured by SeqSim, Pfam similarity, and ECC similarity and the *FS* measures.

### Correlation with gene co-expressions

Genes involved in the same biological process, sharing similar functions or cellular components, tend to exhibit similar expression patterns, so a good correlation ought to exist between co-expressed genes and their *FS*. We used the *S.cerevisiae* gene-expression dataset used in earlier studies^20,21^, which contains co-expression values of 4800 pairs of genes for each ontology, downloaded from GeneMANIA^23^ and other microarray experiments. We computed Pearson’s correlations between gene co-expressions and *FS* values of BP, MF and CC ontologies.

### Correlations with AUC on predicting protein-protein interactions

Two interacting proteins have same CC ontology, share similar functions, and are likely to belong to same BP, so the *FS* between two proteins is an indicative of an interaction^24,25^. The prediction of protein-protein interaction (PPI) was formulated as a classification problem where *FS* exceeding a certain threshold indicated an interaction between two proteins. We used yeast PPI datasets from the Jain and Davis’s database^21,26^, which contain 4385 PPIs on BP, 3858 PPIs on MF, and 4469 PPIs on CC. The human PPIs was downloaded from the Database of Interacting Proteins (DIP)^27^ that contains 1435 PPIs on BP, 1441 PPIs on MF, and 1431 PPIs on CC. The same numbers of negative interactions generated by randomly choosing annotated protein pairs in BP, MF, and CC ontology. The area under the curve (AUC) values of receiver operating characteristic (ROC) curves of the predictor were used to evaluate performances. A ROC curve plots true positive rates (sensitivity) against false positive rates (1-specificity) of prediction at different thresholds.

### Correlations with AUC on predicting disease genes

Recent studies^28,29^ have combined gene-gene similarities, disease gene associations, and disease–disease similarities in order to predict disease associated genes. For example, Zeng *et al*.^28^ used a path-based similarity measure HeteSim^30^ to calculate the similarities between nodes in heterogeneous networks constructed using protein-protein interactions, gene-phenotype associations, and phenotype-phenotype similarity, to prioritize candidate disease genes; and Zou *et al*.^29^ constructed a microRNA-disease network from microRNA–microRNA and disease–disease networks. Inspired by these works, we formulate the prediction of disease associated genes as a *FS* between a known set of disease genes^31^ and the candidate gene. The *FS* between disease-associated genes and the candidate gene were computed using 46 *FS* measures and the performances were evaluated using AUC values of the prediction of the candidate gene as a disease associated gene. The dataset for disease associated gene prediction was constructed from a preliminary set of 78 OMIM (http://www.omim.org) disease phenotypes collected by Schlicker *et al*.^31^. For each of the phenotypes, one disease protein was randomly selected and predicted as a disease candidate by using functional profiles of other genes. Thereby, a disease genes benchmark set consisting of 78 phenotypes and 78 randomly selected known disease proteins as candidates was constructed.

### Significance test for correlation improvement

To determine statistical significance of an improvement of correlations between *FS* and FS* measures and sequence similarities, gene co-expressions, and AUCs of prediction of PPI and disease associated genes, we adopted Williams test^32^ for correlations between two metrics^33,34^. Specifically, to test whether the population correlation between *X*_1_ and *X*_3_ equals the population correlation between *X*_2_ and *X*_3_, we computed the following *t*-test:

1 $$t(n-3)=\frac{({r}_{13}-{r}_{12})\sqrt{(n-1)(1+{r}_{12})}}{\sqrt{\frac{2K(n-1)}{(n-3)}+{\frac{({r}_{23}+{r}_{13})}{4}}^{2}{(1-{r}_{12})}^{3}}}$$

where $$K=1-{{r}_{12}}^{2}-{{r}_{13}}^{2}-{{r}_{23}}^{2}+2{r}_{12}{r}_{13}{r}_{23}$$.

The higher the correlation between the metric scores, the greater the statistical power of this test than the Fisher *r* to *z-*transformation test on independent correlations is. As *FS* and FS* are highly correlated, we used this Williams test^32^ and adopted FDR for multiple test correction.

To determine whether correlations or AUC values are significantly improved for all *FS* measures to FS* measures on each dataset (CESSM, yeast GE, yeast PPI, and the combination of the three datasets), we implemented the Wilcoxon signed rank test with continuity correction^35^, which tests repeated measurements on a single sample to assess whether their population mean ranks differ. This test is suggested as an alternative for *t*-test for dependent samples when the population cannot be assumed to be normally distributed. We used one-sided Wilcoxon signed rank test to show whether FS* significantly improves the performance of *FS* irrespective of the *FS* measure and the type of ontology.

### Funsim measures

#### Information content of a gene ontology term

Gene ontology (GO) is an ontology of terms describing how gene products behave in a cellular context in a species-independent manner and comprises of three ontological domains: biological process (BP), molecular function (MF), and cellular component (CC)^36^: BP is a collection of molecular events, MF defines gene functions in biological processes, and CC describes gene localizations inside a cell. There are three semantic relations between two GO terms: *is-a* is used when one GO term is a subtype of another GO term, *part-of* is used to represent part-whole relationship in the GO terms, and *regulate* is used when the occurrence of one biological process directly affects the manifestation of another process or quality^37^. GO terms and their semantic relations form a hierarchical directed acyclic graph (DAG) where three domains, BP, MF and CC, are represented as the root terms. The ancestor terms in the hierarchy subsume the semantics of descendant terms.

A gene is associated (or annotated) with GO terms describing the properties of its products (i.e., proteins) and with a corpus consisting of GO annotations (GOA) of all genes in an organism. GOA data of species can be readily downloaded from the GO annotation database (http://www.geneontology.org/GO.downloads.annotations.shtml). Functional similarity of a gene pair or a set is determined by the semantic similarities of the GO terms annotating the gene pair or set. Semantic similarity defines a distance between terms in the semantic space of GO and is quantified by the information contents (IC) of the terms. The information content (IC) of a GO term *t* is defined by negative log-likelihood:

2 $$IC(t)=-\,\mathrm{log}\,p(t)$$

where term probability _*P*_(*t*) of term *t* is determined from the annotations of the corpus (corpus-based) or from the structure of the DAG (structure-based). The intuition is that terms in lower levels of DAG, that is, the terms with lower probability carry more specific information than the terms at higher levels in the hierarchy. Corpus-based methods evaluate the term probability as

3 $$p(t)=\frac{M}{N}$$

where *M* is the number of genes annotated by term *t* and *N* is the total number of genes in the annotating corpus.

Structure-based methods evaluate term probability based on the location and the number of children and ancestors of the term. For example, SORA^12^ method defines a term IC as

4 $$IC(t)=depth(t)\,\ast \,(1-\frac{\mathrm{log}(|C(t)|+1)}{\mathrm{log}({T}_{total})})$$

where *depth* (*t*) is the depth and *C* (*t*) is the set of children of term *t* and *T*_*total*_ is the total number of terms in the DAG. The WIS^13^ method extends the idea from SORA^12^ and defines the IC of term *t* not only based on its children but also its depth, as

5 $$IC(t)=depth(t)\,\ast \,\mathrm{log}(|A(t)|)\,\ast \,(1-\frac{\mathrm{log}({\sum }_{x\in C(t)\cup \{t\}}\frac{1}{depth(x)}+1)}{\mathrm{log}({T}_{total})})$$

where *A* (*t*) the set of the ancestor (parent) terms of term *t*. The GO-universal method^8^ combines information from both the corpus and the DAG and defines term probability as

6 $$p(t)=\{\begin{array}{ll}1, & if\,t\,is\,root\\ \prod _{x\in P(t)}\frac{p(x)}{|C(x)|} & otherwise\end{array}$$

#### Semantic similarity measures between GO terms

A semantic similarity measure defines a semantic distance between a pair or a set of GO terms and is evaluated as the IC of the gene pair or set. Since the ancestor terms in the DAG subsume the concepts of descendent terms, semantic similarities are mostly computed based on the ICs of common ancestors of the terms. Let $A(T)=\{{a}_{0},\,{a}_{1},\,\cdots {a}_{n-1}\}$ denote the set of ancestor terms of the GO-terms in the set *T* where *a*_0_ denotes the most informative common ancestor (i.e., the ancestor with the largest IC). The measures by Resnik^6^, Lin^7^, Jiang & Conrath^38^, Nunivers^8^, Schlicker^9^, and XGraSM^10^ (Extended Lin and Nunivers) define semantic similarities or $IC(\{{t}_{1},\,{t}_{2}\})$ of a pair of terms, *t*_1_ and *t*_2_, based on the IC of their most informative common ancestor:

Resnik^6^:

7 $$IC(\{{t}_{1},\,{t}_{2}\})=IC({a}_{0})=\,\max \{IC(x):x\in A(\{{t}_{1},\,{t}_{2}\})\}$$

Lin^7^:

8 $$IC(\{{t}_{1},\,{t}_{2}\})=\frac{2\times IC({a}_{0})}{IC({t}_{1})+IC({t}_{2})}$$

Nunivers^8^:

9 $$IC(\{{t}_{1},{t}_{2}\})\,=\frac{IC({a}_{0})}{{\rm{\max }}\,\{IC({t}_{1}),IC({t}_{2})\}}$$

Zhang^16^:

10 $$IC(\{{t}_{1},\,{t}_{2}\})=\exp (-IC({a}_{0}))$$

Schlicker^9^:

11 $$IC(\{{t}_{1},\,{t}_{2}\})=\frac{2\times IC({a}_{0})}{IC({t}_{1})+IC({t}_{2})}(1-\exp (-IC({a}_{0})))$$

Extended Lin^10^:

12 $$IC(\{{t}_{1},\,{t}_{2}\})=\frac{2\times IC({a}_{0})}{IC({t}_{1})+IC({t}_{2})}\frac{1}{n}(1+{\sum }_{j=1}^{n-1}\frac{IC({a}_{j})}{IC({a}_{0})})$$

Extended Nunivers^10^:

13 $$IC(\{{t}_{1},\,{t}_{2}\})=\frac{IC({a}_{0})}{\max \{IC({t}_{1}),\,IC({t}_{2})\}}\frac{1}{n}(1+{\sum }_{j=1}^{n-1}\frac{IC({a}_{j})}{IC({a}_{0})})$$

All the above similarity measures assign unit weight to every edge in the DAG. Edge-based similarity measures such as Wang^17^, SORA^12^ and WIS^13^ incorporate weights to the edges of the DAG. Wang^17^ defined a semantic value *s*_t_ of term *t* by assigning semantic weight *w* of 0.8 and 0.6 for *is-a* and *part-of* associations, respectively, and

14 $${s}_{t}(x)=\{\begin{array}{ll}1, & if\,x=t\\ {\rm{\max }}\{w(x^{\prime} ):x^{\prime} \in C(x)\}, & otherwise\end{array}$$

And the IC of a term and semantic similarity between terms are computed from semantic values of the ancestors as respectively given by

15 $$IC(t)=\sum _{x\in A(\{t\})\cup \{t\}}{s}_{t}(x)$$

16 $$IC(\{{t}_{1},\,{t}_{2}\})=\frac{{\sum }_{t\in A(\{{t}_{1},{t}_{2}\})}{s}_{{t}_{1}}(t)+{s}_{{t}_{2}}(t)}{IC({t}_{1})+IC({t}_{2})}$$

In methods of SORA^12^ and of WIS^13^, the IC of a term is considered to be composed of an inherited IC from the parent and an extended IC intrinsic to the term; and the extended IC is expressed as a weighted inherited IC from the parent. For a term *t* and its parent *a*, the IC of the pair is given by

17 $$IC(\{a,\,t\})=IC(a)+I{C}_{extended}(a\to t)=IC(a)+IC(t)-wIC(a)$$

where *w* denotes the weight of the association between parent and the term, In SORA^12^ *w* = 1 and in WIS^13^, the weight is computed from the numbers of their children as $w=\frac{|C(t)|}{|C(a)|}$. Eq. (17) provides a means of computing the IC of a given term set without repeatedly summing the shared ICs of ancestors; and SORA and WIS methods define the semantic similarity of a term set *T* as the IC of the term set.

#### Functional similarity measures between two genes

Functional similarity (*FS*) between two genes is computed using the ICs of individual terms (term-based) or the semantic similarities between the pairs of terms (term pair-based) or among the set of terms (term set-based). Let ${T}_{{g}_{1}}$ and ${T}_{{g}_{2}}$ be the set of GO terms annotating genes *g*_1_ and *g*_2_, respectively. Term-based measures such as GIC^1^ (Jaccard index), DIC^39^ (dice index), and UIC^39^ (universal index) are defined using ICs of individual terms:

GIC^1^:

18 $$FS({g}_{1},\,{g}_{2})=\frac{{\sum }_{t\in {T}_{{g}_{1}}\cap {T}_{{g}_{2}}}IC(t)}{{\sum }_{t\in {T}_{{g}_{1}}\cup {T}_{{g}_{2}}}IC(t)}$$

DIC^39^:

19 $$FS({g}_{1},\,{g}_{2})=\frac{2\times {\sum }_{t\in {T}_{{g}_{1}}\cap {T}_{{g}_{2}}}IC(t)}{{\sum }_{t\in {T}_{{g}_{1}}}IC(t)+{\sum }_{t\in {T}_{{g}_{2}}}IC(t)}$$

UIC^39^:

20 $$FS({g}_{1},\,{g}_{2})=\frac{{\sum }_{t\in {T}_{{g}_{1}}\cap {T}_{{g}_{2}}}IC(t)}{{\rm{\max }}\{{\sum }_{t\in {T}_{{g}_{1}}}IC(t),\,{\sum }_{t\in {T}_{{g}_{2}}}IC(t)\}}$$

Term pair-based methods are defined by pairwise gene similarities and use statistical closeness measures such as Average (AVG) and Maximum (MAX), Best-Match Average (BMA) and Average Best-Matches (ABM):

AVG:

21 $$FS({g}_{1},\,{g}_{2})=\frac{1}{|{T}_{{g}_{1}}||{T}_{{g}_{2}}|}\,{\sum }_{{t}_{1}\in {T}_{{g}_{1}},{t}_{2}\in {T}_{{g}_{2}}}IC\,(\{{t}_{1},\,{t}_{2}\})$$

MAX:

22 $$FS({g}_{1},\,{g}_{2})=\,\max \{IC(\{{t}_{1},\,{t}_{2}\}):{t}_{1}\in {T}_{{g}_{1}},\,{t}_{2}\in {T}_{{g}_{2}}\}$$

BMA:

23 $$FS({g}_{1},\,{g}_{2})=\frac{1}{2}(\frac{1}{|{T}_{{g}_{1}}|}\,\sum _{{t}_{1}\in {T}_{{g}_{1}}}IC(\{{t}_{1},\,{t}_{2}\})+\frac{1}{|{T}_{{g}_{2}}|}\,\sum _{{t}_{2}\in {T}_{{g}_{2}}}\,IC(\{{t}_{1},{t}_{2}\}))$$

AMB:

24 $$FS({g}_{1},\,{g}_{2})=\frac{1}{|{T}_{{g}_{1}}||{T}_{{g}_{2}}|}(\sum _{{t}_{1}\in {T}_{{g}_{1}}}IC(\{{t}_{1},\,{t}_{2}\})+\sum _{{t}_{2}\in {T}_{{g}_{2}}}IC(\{{t}_{1},\,{t}_{2}\}))$$

Term set-based measures Overlap Ratio (OR) and Intersection to Union Ratio (IUR) were introduced by SORA^12^ and WIS^13^ methods, respectively, and use ICs of term sets:

OR:

25 $$FS({g}_{1},\,{g}_{2})=\frac{1}{2}(\frac{IC({T}_{{g}_{1}}\cap {T}_{{g}_{2}})}{IC({T}_{{g}_{1}})}+\frac{IC({T}_{{g}_{1}}\cap {T}_{{g}_{2}})}{IC({T}_{{g}_{2}})})$$

IUR:

26 $$FS({g}_{1},\,{g}_{2})=\frac{IC({T}_{{g}_{1}}\cap {T}_{{g}_{2}})}{IC({T}_{{g}_{1}}\cup {T}_{{g}_{2}})}$$

Table 2 lists 46 *FS* measures itemized based on nine corpus-based and two structure-based semantic similarities of GO terms, which are combined using direct-term based measures (DIC, GIC, and UIC), pair-based measures (MAX, AVG, BMA, and ABM), and set-based operations (OR and IUR). There exist several packages implementing some of these measures: for example, GOSemSim^40^, an R package implementing five measures, Resnik, Lin, Jiang, Schlicker and Wang’s similarity, and A-DaGO-Fun^41^, a python package, implementing 44 corpus-based *FS* measures. We extend these works to include structure-based methods such as SORA and WIS and developed EnrichFunSim (https://gitlab.com/liuwt/EnrichFunSim), a python package that implements all 46 *FS* measures and corresponding FS* measures discussed in this paper.

#### Association of a candidate gene with a disease

The association of a candidate gene with a specific disease is computed as the *FS* between a set of known disease associated genes and a candidate gene. Given the set *G*_*d*_ of disease-associated genes of disease *d* and a candidate gene *g*, the association of gene with the disease is given by *FS* (*G*_*d*_, *g*), the functional similarity between *G*_*d*_ and *g*.

#### Enriched Functional Similarity

**Figure 2:**
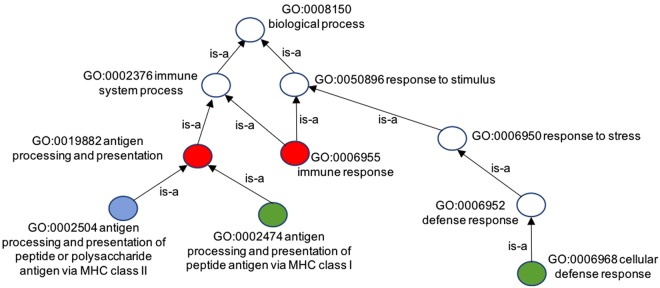
The DAG representing the sets of GO terms annotating proteins: P01906 and P17693. Protein P01906 is annotated by terms *GO*:0006955, *GO*:0019882 and *GO*:0002504; and P17693 is annotated by terms *GO*:0006955, *GO*:0019882, *GO*:0002474 and *GO*:0006968. Blue terms denote terms annotating only protein P01906, green terms denote terms annotating only protein P17693, and red terms denote terms annotating both proteins.

Generally, *FS* measures of genes are derived from ICs of GO terms annotating the genes and the ICs are computed by either how terms are annotated or represented in the corpus or how terms are represented in the DAG. However, how GO terms are represented in the querying gene pair or set has not been considered when evaluating the *FS* of a pair or a set of genes. In other words, the existing FS measures do not consider how GO terms are represented or enriched by the querying pair of genes. The enrichment of a GO term by the pair of gene is dependent on whether the term is annotated by one gene (i.e., partial annotation) or by both genes (i.e. complete annotation) in the pair. For example, consider the protein pair (P01906, P17693) shown in Fig. 2 and the GO DAG of their terms set. The terms *GO*:006955 and *GO*:0019882 are commonly annotated to both proteins; *GO*:0002504 is annotated to only P01906; and *GO*:002474 and *GO*:0006968 are annotated to only P17693. The enrichment of a term by the querying gene pair depends on whether the term annotates one gene or both the genes. Existing *FS* measures does not take note of whether the term annotates only one gene or both genes when computing their ICs.

**Figure 3:**
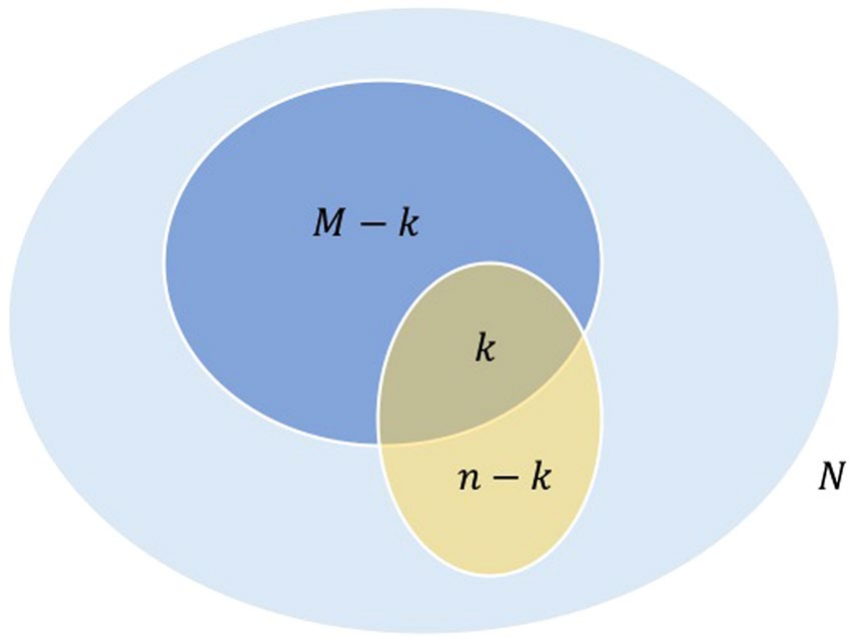
Venn diagram illustrating a GO term annotating the genes in the corpus (blue) and the querying set (yellow): *M* genes in the corpus of *N* genes and *k* genes in a querying set of *n* genes are annotated by the GO term.

We introduce GO term enrichment by the pair of genes in the computation of IC and propose enriched *FS* (FS^*^) between two genes. The probability of term *t* annotating *k* genes in a gene set of size *n* is given by a hypergeometric distribution as

27 $$p(k,\,n|t)=\frac{(\begin{array}{c}M\\ k\end{array})(\begin{array}{c}N-M\\ n-k\end{array})}{(\begin{array}{c}N\\ n\end{array})},\,{\rm{where}}\,k=\{0,\,\cdots ,\,n\}$$

where *N* is the number of genes in the corpus and *M* is the number of genes that annotate term *t*. Figure 3 shows the Venn diagram depicting how a gene is enriched by the annotating corpus and the querying gene set. Note that in (27), the annotation of term *t* by the corpus is represented by set {*N*, *M*} and by the querying pair is by set {*n*, *k*}.

We define the enriched probability term p*(*t*) as the joint probability *p* (*k*, *n*, *t*) of *k* genes in a querying set of *n* genes, being annotated by term *t* as

28 $${p}^{\ast }(t)=p(k,\,n,\,t)=p(k,\,n|t)p(t)$$

and enriched IC, $I{C}^{\ast }(t)=-\,\mathrm{log}({p}^{\ast }(t))$ is given by

29 $$I{C}^{\ast }(t)=-\,\mathrm{log}(p(k,n|t))+IC(t)$$

where *IC* (*t*) is computed from the prior knowledge by annotations of corpus or by the expert knowledge embedded in the DAG. Note that by including {*n*, *k*} in the IC of term, IC^*^ (*t*) accounts for the enrichment of the term by querying gene set.

From (27) and (29), for a querying gene pair (*n* = 2), when term *t* is partially annotated (*k* = 1):

30 $$I{C}^{\ast }(t)=-\,\mathrm{log}(\,\frac{2M(N-M)}{N(N-1)})+IC(t)$$

and when term *t* is fully annotated (*k* = 2):

31 $$I{C}^{\ast }(t)=-\,\mathrm{log}(\frac{M(M-1)}{N(N-1)})+IC(t)$$

By substituting IC^*^ (*t*) for *IC* (*t*) in the computation of *FS* measures of gene pairs, we obtain corresponding enriched FS^*^ measures. The method is applicable to both corpus-based and structure-based methods evaluating IC. It also provides a means for incorporating information of querying gene pair and the annotating corpus in the structure-based methods of evaluating *FS*.

### Software availability

The software (python code) and all the benchmark datasets evaluation (R script) are available at https://gitlab.com/liuwt/EnrichFunSim.

## Electronic supplementary material

Supplementary Information
