# Supplementary material for: Gene Ontology Enrichment Improves Performances of Functional Similarity of Genes
Source: Sci Rep. 2018 Aug 14;8:12100. doi: 10.1038/s41598-018-30455-0 (PMC6092333; doi:10.1038/s41598-018-30455-0)
Supplement: Supplementary file 1 — Supplementary Information [file 41598_2018_30455_MOESM1_ESM.pdf]

Supplementary Materials

Gene Ontology Enrichment Improves Performances of Functional Similarity of Genes

Wenting Liu[1], Jianjun Liu[1], Jagath C. Rajapakse[2]

Supplementary Tables

Supplementary Tables 1-6 show the performance of the improvement of *FS** measures over *FS* measures on various datasets of three ontologies with IEA (Inferred from Electronic Annotation) annotations. In each table, the significant improvement with FDR<0.01 are bolded, and the significant reductions are marked in red; on each dataset, the top *FS* and top *FS** performers are bolded, and the top improvement are marked in green.

Table 1. Improvements of performances of *FS** measures over *FS* measures, using BP ontology on predicting ECC, Pfam, SeqSim similarities of protein pairs of CESSM datasets.

Experiments	ECC_BP				Pfam_BP				SeqSim_BP			
FS measure	*FS*	*FS**	improve - ment(%)	FDR p-value	*FS*	*FS**	improve - ment(%)	FDR p-value	*FS*	*FS**	improve - ment(%)	FDR p-value
U_{ABM}	0.4311	0.4332	**0.49**	**1.89E-07**	**0.4754**	**0.4758**	0.08	2.41E-01	0.6992	0.6968	-0.33	1.46E-14
Z_{ABM}	0.4314	0.4339	**0.58**	**6.74E-69**	0.4363	0.4416	**1.20**	**0.00E+00**	0.6182	0.6228	0.74	**2.10E-320**
W_{ABM}	0.4289	0.4298	0.21	3.99E-01	0.4621	**0.5261**	**13.84**	**1.50E-71**	0.6625	**0.7741**	**16.85**	**0.00E+00**
N_{ABM}	0.4445	0.4466	**0.47**	**5.29E-71**	0.4256	0.4297	**0.96**	**1.87E-273**	0.6393	0.6435	**0.64**	**0.00E+00**
XN_{ABM}	0.4600	0.4605	**0.10**	**4.47E-05**	0.4673	0.4710	**0.78**	**1.25E-236**	0.7227	0.7262	**0.49**	**0.00E+00**
L_{ABM}	0.4290	0.4317	**0.64**	**1.76E-87**	0.3953	0.4000	**1.18**	**4.47E-264**	0.5971	0.6019	**0.81**	**0.00E+00**
XL_{ABM}	0.4547	0.4556	**0.21**	**1.31E-10**	0.4513	0.4556	**0.96**	**4.88E-231**	0.7030	0.7075	**0.64**	**0.00E+00**
S_{ABM}	0.4303	0.4321	**0.42**	**3.97E-36**	0.3943	0.3994	**1.30**	**2.34E-287**	0.5952	0.6011	**0.99**	**0.00E+00**
U_{BMA}	0.4362	0.4383	**0.48**	**6.04E-07**	**0.4764**	**0.4764**	0.00	3.99E-01	0.6883	0.6858	-0.35	4.26E-14
Z_{BMA}	0.4472	0.4491	**0.41**	**5.23E-50**	0.4526	0.4570	**0.96**	**7.83E-278**	0.6288	0.6319	**0.50**	**2.83E-179**
W_{BMA}	0.4388	0.4382	-0.15	3.99E-01	0.4716	**0.5223**	**10.74**	**1.64E-44**	0.6633	**0.7691**	**15.95**	**3.95E-323**
N_{BMA}	0.4635	**0.4651**	**0.33**	**2.25E-51**	0.4373	0.4408	**0.78**	**1.08E-244**	0.6482	0.6513	**0.47**	**1.08E-269**
XN_{BMA}	**0.4748**	**0.4748**	0.00	3.99E-01	0.4721	0.4752	**0.64**	**3.37E-209**	0.7256	0.7283	**0.37**	**2.10E-263**
L_{BMA}	0.4497	0.4518	**0.46**	**6.49E-64**	0.4114	0.4153	**0.95**	**2.50E-216**	0.6094	0.6130	**0.59**	**2.33E-245**
XL_{BMA}	**0.4708**	**0.4748**	**0.84**	**2.53E-253**	0.4590	**0.4752**	**3.53**	**0.00E+00**	0.7091	0.7283	**2.71**	**0.00E+00**
S_{BMA}	0.4511	0.4523	**0.26**	**4.89E-16**	0.4102	0.4147	**1.10**	**4.91E-214**	0.6069	0.6121	**0.86**	**0.00E+00**
U_{MAX}	0.3000	0.3016	0.54	2.70E-02	0.1170	0.1179	0.75	1.87E-01	0.1244	0.1260	1.25	2.68E-02
Z_{MAX}	0.3311	0.3316	**0.15**	**1.07E-12**	0.1289	0.1292	**0.23**	**7.16E-05**	0.1189	0.1191	**0.22**	**8.05E-03**
W_{MAX}	0.3061	0.2964	-3.16	8.23E-03	0.1136	0.1161	2.18	3.24E-01	0.1200	**0.1318**	**9.83**	**1.46E-03**
N_{MAX}	0.3142	0.3148	**0.20**	**2.09E-12**	0.1237	0.1241	**0.36**	**1.12E-05**	0.1209	0.1212	**0.30**	**9.97E-04**
XN_{MAX}	0.3111	0.3115	**0.14**	**2.87E-08**	0.1191	0.1195	**0.33**	**1.07E-07**	0.1287	0.1289	**0.15**	**8.24E-03**
L_{MAX}	0.3089	0.3098	**0.27**	**9.52E-20**	0.1194	0.1199	**0.42**	**2.45E-06**	0.1164	0.1168	**0.34**	**1.69E-04**
XL_{MAX}	0.3144	0.3148	**0.12**	**2.19E-06**	0.1196	0.1199	**0.25**	**8.01E-04**	0.1273	0.1276	**0.17**	**7.10E-04**
S_{MAX}	0.3093	0.3096	0.09	1.16E-02	0.1196	0.1198	0.20	9.57E-02	0.1162	0.1167	**0.49**	**2.98E-05**
U_{AVG}	0.2526	0.2567	**1.60**	**3.06E-13**	0.3248	0.3276	**0.87**	**5.10E-07**	0.3741	0.3764	**0.59**	**2.67E-05**
Z_{AVG}	0.2068	0.2146	**3.77**	**1.20E-231**	0.2897	0.2974	**2.65**	**7.34E-237**	0.2557	0.2650	**3.66**	**0.00E+00**
W_{AVG}	0.2395	0.2379	-0.68	3.99E-01	0.3492	0.3297	-5.56	2.78E-04	0.3627	**0.4386**	**20.93**	**6.59E-54**
N_{AVG}	0.2352	0.2415	**2.67**	**5.03E-181**	0.2790	0.2857	**2.42**	**1.95E-211**	0.2938	0.3025	**2.96**	**0.00E+00**
XN_{AVG}	0.2409	0.2456	**1.96**	**7.80E-75**	0.2770	0.2838	**2.47**	**2.38E-162**	0.3268	0.3365	**2.97**	**0.00E+00**
L_{AVG}	0.2249	0.2316	**2.98**	**2.86E-189**	0.2751	0.2822	**2.60**	**3.97E-220**	0.2743	0.2833	**3.27**	**0.00E+00**
XL_{AVG}	0.2336	0.2389	**2.28**	**1.99E-83**	0.2746	0.2822	**2.75**	**1.45E-178**	0.3109	0.3215	**3.41**	**0.00E+00**
S_{AVG}	0.2328	0.2339	**0.46**	**7.19E-08**	0.2775	0.2820	**1.62**	**7.97E-123**	0.2817	0.2849	**1.13**	**3.55E-62**
D_{DIC}	0.4396	0.4392	-0.08	4.85E-12	0.4545	0.4551	**0.13**	**1.54E-26**	0.7228	0.7246	**0.24**	**0.00E+00**
Z_{DIC}	0.4490	0.4490	0.02	3.99E-01	0.4606	0.4616	**0.22**	**9.12E-89**	0.7167	0.7184	**0.23**	**0.00E+00**
W_{DIC}	0.4553	0.4554	0.04	9.96E-02	0.4490	0.4495	**0.11**	**9.61E-18**	0.6988	0.6997	**0.12**	**2.32E-87**
U_{DIC}	0.4335	0.4390	**1.27**	**2.05E-30**	0.4581	0.4579	-0.05	3.72E-01	0.7281	0.7247	-0.46	1.92E-20
D_{GIC}	0.4175	0.4169	-0.15	1.31E-36	0.4607	0.4608	0.04	4.19E-02	**0.7622**	**0.7633**	**0.14**	**1.21E-248**
Z_{GIC}	0.4263	0.4261	-0.06	1.04E-05	0.4665	0.4670	**0.11**	**4.94E-32**	**0.7592**	**0.7603**	**0.15**	**7.71E-293**
W_{GIC}	0.4323	0.4321	-0.04	6.92E-05	0.4588	0.4590	**0.03**	**5.28E-05**	0.7446	0.7450	**0.06**	**1.40E-29**
U_{GIC}	0.4096	0.4143	**1.16**	**5.61E-31**	0.4576	0.4585	0.19	3.26E-02	0.7584	0.7570	-0.19	2.98E-06
D_{UIC}	0.4212	0.4231	0.44	3.32E-01	0.4484	0.4290	-4.33	5.46E-15	0.7284	0.6902	-5.24	1.65E-92
Z_{UIC}	0.4288	0.4310	0.52	3.16E-01	0.4538	0.4332	-4.53	1.43E-15	0.7237	0.6807	-5.93	8.21E-106
W_{UIC}	0.4327	0.4375	1.10	8.03E-02	0.4427	0.4207	-4.97	2.53E-17	0.7074	0.6623	-6.37	2.29E-110
U_{UIC}	0.4141	0.4277	**3.27**	**1.82E-13**	0.4532	0.4490	-0.91	2.74E-02	0.7368	0.7126	-3.28	3.53E-72
R_{OR}	0.4421	0.4575	**3.49**	3.99E-01	0.4933	0.5199	**5.38**	**3.63E-02**	0.7884	0.7738	-1.85	1.10E-03
I_{UR}	0.3990	0.4147	**3.94**	3.99E-01	0.4728	0.5005	**5.86**	**3.36E-02**	0.7927	0.8028	**1.28**	**6.67E-03**

Table 2. Improvements of performances of *FS** measures over *FS* measures, using MF ontology on predicting ECC, Pfam, SeqSim similarities of protein pairs of CESSM datasets

Experiments	ECC_MF				Pfam_MF				SeqSim_MF			
Methods	*FS*	*FS**	improve - ment(%)	FDR p-value	*FS*	*FS**	improve - ment(%)	FDR p-value	*FS*	*FS**	improve - ment(%)	FDR p-value
U_{ABM}	0.6973	0.6981	**0.11**	**2.96E-39**	0.5128	0.5123	-0.10	1.09E-12	0.4127	0.4123	-0.10	1.24E-07
Z_{ABM}	0.6928	0.6954	**0.37**	**4.67E-81**	0.4813	0.4874	**1.28**	**0.00E+00**	0.3874	0.3939	**1.68**	**0.00E+00**
W_{ABM}	0.6889	**0.7479**	**8.56**	**2.13E-152**	0.5034	**0.6283**	**24.82**	**0.00E+00**	0.4050	**0.5425**	**33.95**	**0.00E+00**
N_{ABM}	0.7248	0.7294	**0.64**	**1.26E-243**	0.5259	0.5309	**0.95**	**2.69E-185**	0.4386	0.4419	**0.77**	**2.38E-71**
XN_{ABM}	0.7292	0.7337	**0.61**	**0.00E+00**	**0.5829**	0.5849	**0.34**	**5.17E-49**	0.5049	0.5058	**0.18**	**1.51E-09**
L_{ABM}	0.6918	0.6988	**1.02**	**1.86E-277**	0.4794	0.4856	**1.29**	**1.10E-140**	0.3958	0.3997	**0.98**	**1.39E-50**
XL_{ABM}	0.7114	0.7177	**0.88**	**0.00E+00**	0.5650	0.5673	**0.42**	**1.16E-31**	0.4922	0.4931	**0.18**	**3.25E-05**
S_{ABM}	0.6955	0.6986	**0.45**	**7.69E-40**	0.4712	0.4809	**2.06**	**3.04E-259**	0.3869	0.3954	**2.20**	**1.21E-179**
U_{BMA}	0.7152	0.7157	**0.07**	**1.11E-18**	0.5061	0.5051	-0.19	3.91E-51	0.4055	0.4046	-0.22	4.75E-37
Z_{BMA}	0.7145	0.7171	**0.36**	**1.40E-90**	0.4758	0.4821	**1.32**	**0.00E+00**	0.3812	0.3878	**1.73**	**0.00E+00**
W_{BMA}	0.7100	**0.7665**	**7.96**	**2.59E-157**	0.5003	**0.6260**	**25.11**	**0.00E+00**	0.4001	0.5370	**34.21**	**0.00E+00**
N_{BMA}	**0.7485**	**0.7525**	**0.54**	**7.68E-226**	0.5240	0.5282	**0.81**	**5.94E-148**	0.4348	0.4374	**0.61**	**1.83E-50**
XN_{BMA}	**0.7525**	**0.7567**	**0.55**	**0.00E+00**	0.5818	0.5833	**0.27**	**3.68E-30**	0.5017	0.5021	**0.08**	**7.50E-03**
L_{BMA}	0.7176	0.7238	**0.87**	**1.58E-260**	0.4794	0.4846	**1.09**	**2.16E-110**	0.3939	0.3969	**0.75**	**1.02E-33**
XL_{BMA}	**0.7362**	0.7421	**0.80**	**0.00E+00**	0.5655	0.5673	**0.32**	**3.34E-21**	0.4910	0.4912	0.04	2.70E-01
S_{BMA}	0.7189	0.7229	**0.56**	**1.50E-75**	0.4692	0.4794	**2.17**	**6.00E-305**	0.3829	0.3920	**2.38**	**2.24E-218**
U_{MAX}	0.3974	0.3971	-0.07	5.98E-06	0.1642	0.1641	-0.06	1.35E-01	0.0876	0.0875	-0.07	1.49E-01
Z_{MAX}	0.3835	0.3845	**0.24**	**6.69E-03**	0.1590	0.1593	0.15	2.92E-01	0.0845	0.0848	0.37	3.01E-01
W_{MAX}	0.3923	0.4047	**3.15**	**1.97E-10**	0.1615	0.1670	3.43	1.13E-02	0.0862	0.0893	3.60	1.49E-01
N_{MAX}	0.3875	0.3881	**0.15**	**1.94E-19**	0.1612	0.1614	**0.16**	**7.52E-03**	0.0859	0.0860	0.16	1.67E-01
XN_{MAX}	0.3974	0.3977	0.07	8.54E-02	0.1648	0.1649	0.05	3.43E-01	0.0879	0.0880	0.04	3.52E-01
L_{MAX}	0.3098	0.3824	**23.44**	**0.00E+00**	0.1199	0.1587	**32.39**	**0.00E+00**	0.1168	0.0845	-27.71	0.00E+00
XL_{MAX}	0.3943	0.3947	0.09	7.99E-02	0.1626	0.1628	0.13	2.75E-01	0.0871	0.0872	0.11	3.73E-01
S_{MAX}	0.3819	0.3823	0.12	2.84E-01	0.1593	0.1590	-0.18	3.59E-01	0.0842	0.0844	0.21	3.86E-01
U_{AVG}	0.4070	0.4131	**1.50**	**0.00E+00**	0.4817	0.4825	**0.17**	**1.53E-07**	0.4508	0.4489	-0.43	1.82E-36
Z_{AVG}	0.5101	0.5091	-0.20	4.85E-10	0.4492	0.4527	**0.77**	**4.30E-106**	0.3494	0.3515	**0.62**	**6.05E-35**
W_{AVG}	0.4090	0.3943	-3.59	1.95E-03	0.4788	0.5238	**9.41**	**1.81E-26**	0.4313	**0.5514**	**27.84**	**1.42E-184**
N_{AVG}	0.4792	0.4956	**3.42**	**0.00E+00**	0.4678	0.4750	**1.54**	**1.18E-140**	0.3687	0.3768	**2.21**	**3.02E-161**
XN_{AVG}	0.4556	0.4791	**5.16**	**0.00E+00**	0.4916	0.5043	**2.59**	**1.17E-250**	0.4046	0.4182	**3.35**	**1.44E-260**
L_{AVG}	0.4547	0.4728	**3.97**	**0.00E+00**	0.4449	0.4516	**1.50**	**1.21E-97**	0.3418	0.3487	**2.03**	**7.40E-94**
XL_{AVG}	0.4267	0.4532	**6.21**	**0.00E+00**	0.4681	0.4817	**2.90**	**1.82E-235**	0.3768	0.3905	**3.64**	**2.38E-216**
S_{AVG}	0.4701	0.4746	**0.96**	**1.15E-51**	0.4435	0.4491	**1.27**	**2.90E-77**	0.3437	0.3466	**0.84**	**2.06E-19**
D_{DIC}	0.6653	0.6671	**0.28**	**8.60E-39**	0.5710	0.5769	**1.03**	**0.00E+00**	0.5288	0.5375	**1.64**	**0.00E+00**
Z_{DIC}	0.5978	0.5970	-0.13	8.33E-51	0.4506	0.4503	-0.07	2.63E-07	0.4046	0.4061	**0.36**	**4.84E-154**
W_{DIC}	0.5958	0.5942	-0.26	3.14E-130	0.4261	0.4231	-0.72	0.00E+00	0.3501	0.3475	-0.74	1.15E-285
U_{DIC}	0.5478	0.5468	-0.18	2.78E-256	0.3370	0.3352	-0.53	0.00E+00	0.2831	0.2818	-0.45	0.00E+00
D_{GIC}	0.6448	0.6450	0.02	2.37E-01	**0.6170**	**0.6203**	**0.53**	**2.08E-124**	**0.6285**	**0.6358**	**1.17**	**0.00E+00**
Z_{GIC}	0.5985	0.5975	-0.16	7.85E-75	0.5224	0.5219	-0.09	3.66E-18	0.5127	0.5146	**0.36**	**1.45E-255**
W_{GIC}	0.6053	0.6041	-0.21	2.39E-70	0.5028	0.4999	-0.58	0.00E+00	0.4481	0.4454	-0.61	7.08E-270
U_{GIC}	0.5580	0.5569	-0.19	6.20E-256	0.4036	0.4016	-0.49	0.00E+00	0.3668	0.3652	-0.44	0.00E+00
D_{UIC}	0.6183	0.6183	0.00	3.99E-01	0.5729	0.5729	0.00	3.99E-01	0.5510	0.5510	0.00	3.99E-01
Z_{UIC}	0.5608	0.5474	-2.38	8.59E-05	0.4622	0.3957	-14.38	6.21E-80	0.4317	0.3689	-14.56	4.07E-69
W_{UIC}	0.5684	0.5477	-3.63	9.67E-12	0.4456	0.3715	-16.63	2.20E-116	0.3786	0.3171	-16.25	1.51E-75
U_{UIC}	0.5283	0.5210	-1.38	7.22E-03	0.3538	0.3052	-13.72	9.53E-66	0.3081	0.2641	-14.28	2.66E-52
R_{OR}	0.7406	0.7828	**5.69**	**1.78E-19**	0.6565	0.6829	**4.03**	**1.44E-03**	0.6505	0.6231	-4.22	3.27E-03
I_{UR}	0.6726	0.7071	**5.13**	**3.24E-06**	0.6627	0.6961	**5.04**	**1.21E-05**	0.7165	0.7217	0.72	3.99E-01

Table 3. Improvements of performances of *FS** measures over *FS* measures predicting gene co-expressions on yeast GE dataset of BP, MF, CC ontologies

Experiments	GE_BP				GE_MF				GE_CC			
Methods	*FS*	*FS**	improve-ment(%)	FDR p-value	*FS*	*FS**	improve-ment(%)	FDR p-value	*FS*	*FS**	improve-ment(%)	FDR p-value
U_{ABM}	**0.2826**	**0.2855**	**1.00**	**1.05E-02**	0.1886	0.1902	**0.88**	**9.67E-07**	0.394	0.3997	**1.45**	**1.24E-06**
Z_{ABM}	0.2207	0.2249	**1.92**	**3.07E-28**	0.1746	0.1754	0.47	**5.11E-02**	0.3808	0.3839	**0.82**	**1.00E-23**
W_{ABM}	0.2532	0.2603	**2.81**	2.95E-01	0.1648	0.1822	**10.53**	**1.56E-02**	0.3912	0.3878	-0.87	3.64E-01
N_{ABM}	0.2392	0.2426	**1.42**	**8.40E-22**	0.1811	0.1835	**1.36**	**1.45E-05**	0.3866	0.3889	**0.61**	**4.88E-20**
XN_{ABM}	0.2532	0.2559	**1.07**	**9.47E-12**	0.1886	0.1902	**0.88**	**6.69E-03**	0.3927	0.3938	**0.28**	**4.49E-08**
L_{ABM}	0.2243	0.2281	**1.67**	**1.29E-24**	0.1707	0.1734	**1.59**	**1.01E-05**	0.3712	0.3742	**0.8**	**1.77E-24**
XL_{ABM}	0.2399	0.2431	**1.34**	**3.58E-14**	0.1789	0.1809	**1.12**	**4.36E-03**	0.3863	0.3877	**0.38**	**1.27E-09**
S_{ABM}	0.2273	0.2288	**0.66**	**4.15E-08**	0.1799	0.1762	-2.02	6.15E-06	0.3741	0.3741	-0.02	3.99E-01
U_{BMA}	**0.28**	**0.2828**	**0.99**	**1.25E-02**	0.1886	0.1902	**0.85**	**9.67E-07**	0.3781	0.3833	**1.4**	**1.09E-05**
Z_{BMA}	0.218	0.2222	**1.91**	**2.36E-28**	0.1759	0.1767	0.49	**5.85E-02**	0.3663	0.3693	**0.82**	**1.14E-21**
W_{BMA}	0.2508	0.2596	**3.50**	2.59E-01	0.1652	0.1841	**11.42**	**8.78E-03**	0.3775	0.3698	-2.03	2.53E-01
N_{BMA}	0.2371	0.2405	**1.43**	**7.74E-22**	0.1824	0.1849	**1.36**	**6.51E-06**	0.3711	0.3733	**0.59**	**8.26E-18**
XN_{BMA}	0.2524	0.2551	**1.06**	**5.51E-12**	0.1905	0.1921	**0.85**	**6.18E-03**	0.3753	0.3762	**0.25**	**6.61E-06**
L_{BMA}	0.2224	0.2261	**1.67**	**2.49E-23**	0.1721	0.1749	**1.63**	**6.51E-06**	0.357	0.3598	**0.79**	**2.69E-21**
XL_{BMA}	0.2397	0.2428	**1.31**	**1.42E-13**	0.1812	0.1832	**1.12**	**4.36E-03**	0.3699	0.3711	**0.32**	**9.81E-08**
S_{BMA}	0.2252	0.2268	**0.70**	**1.48E-08**	0.181	0.1776	-1.85	2.82E-05	0.3598	0.3595	-0.07	3.12E-01
U_{MAX}	0.2299	0.2313	0.58	2.63E-01	0.1385	0.1396	**0.76**	**2.17E-03**	0.2648	0.2658	0.38	3.07E-01
Z_{MAX}	0.1825	0.1855	**1.67**	**6.99E-16**	0.1335	0.1338	0.25	3.55E-01	0.242	0.2444	**0.97**	**3.49E-10**
W_{MAX}	0.2111	0.2145	**1.60**	3.69E-01	0.124	0.144	**16.17**	**8.78E-03**	0.2587	0.2669	**3.15**	2.48E-01
N_{MAX}	0.1928	0.1955	**1.41**	**6.09E-14**	0.1378	0.1393	**1.11**	**8.41E-03**	0.2479	0.2496	**0.67**	**7.65E-09**
XN_{MAX}	0.2164	0.2179	**0.71**	**3.83E-05**	0.1482	0.1488	0.39	2.24E-01	0.2656	0.2662	**0.21**	**1.99E-03**
L_{MAX}	0.1827	0.1855	**1.53**	**1.45E-12**	0.1318	0.1335	**1.29**	**7.35E-03**	0.2379	0.2399	**0.85**	**7.75E-09**
XL_{MAX}	0.2097	0.2117	**0.93**	**7.73E-07**	0.1437	0.1443	0.45	2.61E-01	0.2649	0.2653	0.17	**6.09E-02**
S_{MAX}	0.184	0.1859	**1.04**	**4.79E-10**	0.1382	0.1354	-2.04	4.36E-03	0.2404	0.2396	-0.35	1.35E-01
U_{AVG}	0.2609	0.2694	**3.26**	**1.06E-05**	0.1744	0.1753	0.55	**5.82E-03**	0.1869	0.2062	**10.34**	**1.73E-30**
Z_{AVG}	0.1972	0.2024	**2.67**	**7.40E-31**	0.1698	0.1702	0.26	2.65E-01	0.2673	0.27	**1.02**	**9.66E-11**
W_{AVG}	0.2282	0.1872	-17.96	1.17E-03	0.1608	0.1408	-12.45	1.82E-02	0.2483	0.1422	-42.73	2.26E-20
N_{AVG}	0.2113	0.2159	**2.17**	**7.03E-27**	0.1736	0.1749	0.77	**4.20E-02**	0.254	0.2564	**0.93**	**5.60E-10**
XN_{AVG}	0.2067	0.2109	**2.03**	**1.60E-16**	0.1729	0.1737	0.42	2.37E-01	0.2119	0.2118	-0.05	3.92E-01
L_{AVG}	0.2024	0.207	**2.25**	**7.03E-27**	0.1712	0.1729	**0.98**	**1.61E-02**	0.2508	0.253	**0.88**	**4.93E-08**
XL_{AVG}	0.1945	0.1985	**2.05**	**2.18E-15**	0.1706	0.1717	0.66	1.84E-01	0.2126	0.2122	-0.14	2.80E-01
S_{AVG}	0.2071	0.2082	**0.55**	**1.17E-03**	0.177	0.1747	-1.28	6.18E-03	0.2572	0.254	-1.22	5.60E-10
D_{DIC}	0.2829	0.2839	**0.35**	**2.20E-09**	**0.2002**	**0.2008**	**0.26**	**1.84E-02**	0.422	0.4229	**0.21**	**2.53E-07**
Z_{DIC}	0.2798	0.2809	**0.40**	**1.32E-13**	0.1839	0.1838	-0.06	3.68E-01	**0.4253**	**0.4263**	**0.24**	**8.32E-09**
W_{DIC}	0.2773	0.2784	**0.37**	**2.74E-10**	0.1832	0.1831	-0.07	3.55E-01	0.4063	0.4064	0.04	3.12E-01
U_{DIC}	**0.2812**	**0.2854**	**1.50**	**6.73E-04**	0.1743	0.174	-0.13	4.36E-03	0.392	0.398	**1.55**	**9.21E-10**
D_{GIC}	**0.2873**	**0.2877**	**0.14**	**6.90E-03**	**0.2022**	**0.2023**	**0.05**	3.69E-01	0.4202	0.4204	0.05	1.89E-01
Z_{GIC}	**0.2869**	**0.2875**	**0.20**	**2.45E-06**	0.1885	0.1881	-0.2	**2.24E-02**	**0.4233**	**0.4236**	**0.07**	**5.59E-02**
W_{GIC}	0.2839	0.2843	**0.15**	**8.14E-03**	0.1898	0.1894	-0.21	6.39E-03	0.4081	0.4077	-0.1	8.64E-04
U_{GIC}	0.2762	0.2815	**1.93**	**4.43E-06**	0.1752	0.1748	-0.22	9.67E-07	0.3806	0.3872	**1.74**	**1.47E-12**
D_{UIC}	0.2851	0.2774	-2.69	**5.28E-02**	0.1957	0.1973	**0.81**	3.69E-01	0.419	0.4158	-0.76	3.12E-01
Z_{UIC}	0.2824	0.2747	-2.74	**5.35E-02**	0.18	0.1784	-0.89	3.69E-01	0.4229	0.4189	-0.95	2.89E-01
W_{UIC}	0.277	0.2711	-2.11	1.19E-01	0.1806	0.1764	-2.34	2.37E-01	0.4037	0.3932	-2.59	4.92E-02
U_{UIC}	0.2777	0.276	-0.60	3.52E-01	0.1704	0.17	-0.24	3.96E-01	0.3842	0.3902	**1.59**	1.43E-01
R_{OR}	0.2876	0.2927	**1.77**	**1.00E-01**	0.2087	0.2138	**2.46**	**3.60E-02**	0.3903	0.4103	**5.11**	**1.62E-15**
I_{IUR}	0.2605	0.2736	**5.02**	**9.62E-08**	0.1882	0.1996	**6.07**	**1.02E-03**	0.3821	0.4049	**5.97**	**5.74E-20**

Table 4. Improvements of performances of *FS** measures over *FS* measures predicting protein-protein interactions on human PPI dataset of BP, MF, CC ontologies

Experiments	PPI_BP				PPI_MF				PPI_CC			
Methods	*FS*	*FS**	improve-ment(%)	FDR p-value	*FS*	*FS**	improve-ment(%)	FDR p-value	*FS*	*FS**	improve-ment(%)	FDR p-value
U_{ABM}	0.8521	0.8553	**0.37**	**1.87E-06**	0.7647	0.7650	0.04	1.03E-01	0.7657	0.7658	0.01	3.99E-01
Z_{ABM}	0.8647	0.8652	0.05	4.28E-02	0.7649	0.7648	-0.01	4.06E-01	0.7441	0.7455	**0.19**	**1.51E-04**
W_{ABM}	0.8552	0.8144	**-4.77**	**9.12E-14**	0.7688	0.7223	**-6.05**	**2.05E-12**	0.7646	0.7775	1.69	1.49E-01
N_{ABM}	0.8645	0.8649	0.04	4.78E-02	0.7718	0.7699	**-0.24**	**5.32E-06**	0.7478	0.7493	**0.20**	**6.94E-06**
XN_{ABM}	0.8552	0.8555	0.03	1.82E-01	0.7556	0.7537	**-0.25**	**1.17E-07**	0.7538	0.7551	**0.17**	**5.41E-05**
L_{ABM}	0.8625	0.8630	0.06	2.31E-02	0.7777	0.7762	**-0.19**	**6.23E-04**	0.7437	0.7455	**0.24**	**9.56E-07**
XL_{ABM}	0.8524	0.8529	0.06	4.78E-02	0.7616	0.7598	**-0.24**	**7.72E-06**	0.7536	0.7550	**0.18**	**1.36E-04**
S_{ABM}	0.8628	0.8630	0.03	1.72E-01	0.7787	0.7771	**-0.21**	**6.23E-04**	0.7441	0.7449	0.11	7.81E-02
U_{BMA}	0.8552	0.8580	**0.32**	**6.87E-05**	0.7523	0.7526	0.05	7.62E-02	0.7588	0.7589	0.02	3.99E-01
Z_{BMA}	0.8747	0.8750	0.03	1.72E-01	0.7515	0.7519	0.05	2.77E-01	0.7427	0.7439	**0.17**	**7.35E-04**
W_{BMA}	0.8617	0.8173	**-5.14**	**1.92E-16**	0.7548	0.7213	**-4.44**	**3.90E-07**	0.7598	0.7697	1.31	2.54E-01
N_{BMA}	0.8737	0.8739	0.02	2.30E-01	0.7583	0.7566	**-0.23**	**1.82E-05**	0.7450	0.7463	**0.18**	**7.65E-05**
XN_{BMA}	0.8611	0.8613	0.02	2.96E-01	0.7471	0.7453	**-0.24**	**3.03E-07**	0.7478	0.7488	**0.13**	**1.76E-03**
L_{BMA}	0.8718	0.8721	0.04	1.24E-01	0.7629	0.7614	**-0.21**	**3.71E-04**	0.7424	0.7438	**0.20**	**7.87E-05**
XL_{BMA}	0.8583	0.8587	0.04	1.26E-01	0.7513	0.7495	**-0.24**	**8.12E-06**	0.7485	0.7497	**0.17**	**5.41E-04**
S_{BMA}	0.8721	0.8721	0.00	3.99E-01	0.7643	0.7624	**-0.26**	**3.26E-05**	0.7429	0.7434	0.07	2.49E-01
U_{MAX}	0.8317	0.8326	0.10	2.46E-01	0.6920	0.6921	0.02	4.06E-01	0.6814	0.6811	-0.05	3.99E-01
Z_{MAX}	0.8345	0.8345	0.00	3.99E-01	0.6922	0.6921	-0.01	4.06E-01	0.6812	0.6813	0.00	3.99E-01
W_{MAX}	0.8324	0.8104	**-2.64**	**1.10E-03**	0.6923	0.6890	-0.47	4.06E-01	0.6828	0.6952	1.82	1.81E-01
N_{MAX}	0.8327	0.8328	0.01	3.99E-01	0.6924	0.6925	0.01	4.06E-01	0.6813	0.6814	0.02	3.99E-01
XN_{MAX}	0.8296	0.8296	0.00	3.99E-01	0.6919	0.6920	0.02	4.06E-01	0.6825	0.6828	0.05	3.10E-01
L_{MAX}	0.8326	0.8327	0.01	3.99E-01	0.6928	0.6928	0.00	4.06E-01	0.6821	0.6823	0.03	3.99E-01
XL_{MAX}	0.8288	0.8289	0.01	3.99E-01	0.6922	0.6923	0.02	4.06E-01	0.6844	0.6846	0.03	3.99E-01
S_{MAX}	0.8327	0.8327	0.00	3.99E-01	0.6928	0.6928	-0.01	4.06E-01	0.6814	0.6818	0.06	3.99E-01
U_{AVG}	0.7340	0.7444	**1.42**	**2.48E-07**	0.6722	0.6741	**0.29**	**2.85E-12**	0.6729	0.6740	0.17	3.99E-01
Z_{AVG}	0.6954	0.7012	**0.82**	**1.64E-32**	0.6499	0.6520	**0.32**	**9.50E-05**	0.6125	0.6156	**0.51**	**1.22E-09**
W_{AVG}	0.6943	0.5994	**-13.67**	**8.49E-15**	0.6540	0.5919	**-9.50**	**5.55E-11**	0.6561	0.6790	3.49	2.03E-01
N_{AVG}	0.7312	0.7367	**0.76**	**2.17E-38**	0.6890	0.6852	**-0.54**	**2.34E-07**	0.6227	0.6264	**0.60**	**2.91E-13**
XN_{AVG}	0.7229	0.7290	**0.83**	**1.14E-28**	0.6734	0.6689	**-0.67**	**2.70E-08**	0.6322	0.6370	**0.76**	**6.56E-14**
L_{AVG}	0.7184	0.7241	**0.79**	**8.77E-39**	0.6876	0.6834	**-0.62**	**1.64E-08**	0.6145	0.6180	**0.57**	**5.20E-12**
XL_{AVG}	0.7083	0.7144	**0.87**	**5.72E-29**	0.6729	0.6677	**-0.78**	**1.12E-08**	0.6218	0.6266	**0.77**	**6.56E-14**
S_{AVG}	0.7245	0.7252	0.09	3.48E-02	0.6928	0.6869	**-0.84**	**1.44E-09**	0.6190	0.6192	0.03	3.99E-01
D_{DIC}	0.8523	0.8529	**0.07**	**5.44E-18**	0.7324	0.7320	-0.04	9.72E-02	0.7283	0.7291	**0.11**	**8.24E-05**
Z_{DIC}	0.8528	0.8534	**0.07**	**1.50E-20**	0.7235	0.7248	**0.18**	**4.98E-19**	0.7276	0.7282	**0.09**	**1.83E-03**
W_{DIC}	0.8464	0.8468	**0.05**	**1.72E-07**	0.7021	0.7024	0.03	9.72E-02	0.7221	0.7224	0.05	4.04E-02
U_{DIC}	0.8466	0.8504	**0.45**	**3.10E-07**	0.6964	0.6974	**0.14**	**6.23E-35**	0.7178	0.7224	**0.65**	**3.69E-04**
D_{GIC}	0.8523	0.8529	**0.07**	**3.13E-29**	0.7324	0.7320	-0.04	3.82E-02	0.7283	0.7291	**0.11**	**9.04E-07**
Z_{GIC}	0.8528	0.8534	**0.07**	**4.97E-35**	0.7235	0.7248	**0.18**	**4.88E-34**	0.7276	0.7282	**0.09**	**9.88E-05**
W_{GIC}	0.8464	0.8468	**0.05**	**3.79E-13**	0.7021	0.7024	0.03	4.03E-02	0.7220	0.7224	0.05	1.09E-02
U_{GIC}	0.8465	0.8504	**0.45**	**5.92E-10**	0.6964	0.6974	**0.14**	**6.75E-60**	0.7178	0.7224	**0.65**	**2.65E-05**
D_{UIC}	0.8407	0.8348	-0.70	1.25E-01	0.7361	0.7340	-0.29	4.06E-01	0.7255	0.7211	-0.61	3.87E-01
Z_{UIC}	0.8413	0.8357	-0.67	1.39E-01	0.7261	0.7255	-0.09	4.06E-01	0.7246	0.7199	-0.64	3.85E-01
W_{UIC}	0.8357	0.8306	-0.61	1.72E-01	0.7011	0.7019	0.12	4.06E-01	0.7180	0.7125	-0.77	3.62E-01
U_{UIC}	0.8418	0.8420	0.03	3.99E-01	0.6929	0.6944	0.21	4.06E-01	0.7146	0.7155	0.12	3.99E-01
R_{OR}	0.8298	0.8319	0.25	3.84E-02	0.7507	0.7517	0.13	4.06E-01	0.7452	0.7543	**1.23**	**5.59E-03**
I_{UR}	0.8272	0.8298	0.32	4.78E-02	0.7563	0.7583	0.25	4.06E-01	0.7576	0.7678	**1.35**	**8.98E-04**

Table 5. Improvements of performances of *FS** measures over *FS* measures predicting protein-protein interactions on yeast PPI dataset of BP, MF, CC ontologies

Experiments	PPI_BP				PPI_MF				PPI_CC			
Methods	*FS*	*FS**	improve-ment(%)	FDR p-value	*FS*	*FS**	improve-ment(%)	FDR p-value	*FS*	*FS**	improve-ment(%)	FDR p-value
U_{ABM}	0.8496	0.8499	0.03	3.61E-01	0.6852	0.6853	0.02	2.39E-01	0.8048	0.8072	**0.30**	**9.28E-06**
Z_{ABM}	0.8348	0.8358	**0.12**	**1.72E-18**	0.6790	0.6784	**-0.09**	**6.00E-03**	0.8039	0.8050	**0.13**	**9.07E-15**
W_{ABM}	0.8366	0.8422	0.66	5.61E-02	0.6801	0.6748	-0.78	2.08E-01	0.8014	0.8007	-0.08	4.06E-01
N_{ABM}	0.8461	0.8472	**0.12**	**1.13E-28**	0.6744	0.6761	**0.26**	**2.70E-13**	0.8058	0.8068	**0.13**	**3.82E-21**
XN_{ABM}	0.8518	0.8526	**0.10**	**4.23E-20**	0.6706	0.6726	**0.31**	**9.14E-20**	0.8046	0.8052	**0.07**	**6.32E-12**
L_{ABM}	0.8418	0.8430	**0.14**	**1.08E-28**	0.6693	0.6714	**0.32**	**5.87E-15**	0.7991	0.8003	**0.14**	**8.38E-18**
XL_{ABM}	0.8485	0.8494	**0.11**	**1.62E-18**	0.6661	0.6685	**0.36**	**1.26E-17**	0.8004	0.8011	**0.09**	**7.69E-13**
S_{ABM}	0.8426	0.8431	**0.07**	**1.16E-11**	0.6742	0.6726	**-0.23**	**1.23E-05**	0.8005	0.8000	-0.05	1.03E-02
U_{BMA}	0.8509	0.8513	0.04	3.40E-01	0.6846	0.6847	0.01	4.04E-01	0.8008	0.8036	**0.35**	**1.63E-07**
Z_{BMA}	0.8403	0.8410	**0.09**	**3.04E-12**	0.6765	0.6761	-0.06	1.01E-01	0.8038	0.8049	**0.13**	**5.95E-14**
W_{BMA}	0.8396	0.8416	0.24	3.61E-01	0.6787	0.6737	-0.74	2.31E-01	0.7993	0.7958	-0.44	2.51E-01
N_{BMA}	0.8507	0.8515	**0.09**	**2.38E-18**	0.6769	0.6782	**0.19**	**3.06E-07**	0.8054	0.8064	**0.12**	**9.62E-17**
XN_{BMA}	0.8559	0.8565	**0.07**	**6.63E-13**	0.6719	0.6737	**0.28**	**2.55E-16**	0.8003	0.8008	**0.06**	**5.61E-09**
L_{BMA}	0.8469	0.8478	**0.11**	**2.95E-20**	0.6726	0.6742	**0.24**	**1.91E-08**	0.7996	0.8007	**0.14**	**4.61E-18**
XL_{BMA}	0.8533	0.8540	**0.08**	**1.54E-12**	0.6682	0.6702	**0.30**	**4.14E-12**	0.7966	0.7972	**0.07**	**2.60E-09**
S_{BMA}	0.8473	0.8479	**0.07**	**5.47E-11**	0.6770	0.6754	**-0.24**	**5.39E-06**	0.8013	0.8006	**-0.09**	**1.73E-05**
U_{MAX}	0.8485	0.8487	0.02	3.95E-01	0.6854	0.6853	-0.01	4.04E-01	0.7290	0.7290	0.00	4.06E-01
Z_{MAX}	0.8537	0.8538	0.01	3.92E-01	0.6783	0.6778	-0.07	9.45E-02	0.7272	0.7272	-0.01	4.06E-01
W_{MAX}	0.8467	0.8365	**-1.20**	**4.60E-04**	0.6791	0.6664	**-1.86**	**4.00E-03**	0.7259	0.7280	0.28	3.84E-01
N_{MAX}	0.8542	0.8545	**0.03**	**5.91E-03**	0.6678	0.6681	0.04	3.11E-01	0.7260	0.7262	0.02	2.54E-01
XN_{MAX}	0.8559	0.8563	**0.04**	**3.47E-05**	0.6618	0.6623	0.07	6.98E-02	0.7260	0.7261	0.01	2.62E-01
L_{MAX}	0.8552	0.8554	0.03	4.87E-02	0.6681	0.6683	0.04	3.54E-01	0.7257	0.7257	0.00	4.06E-01
XL_{MAX}	0.8559	0.8561	**0.03**	**7.53E-03**	0.6605	0.6614	**0.13**	**9.28E-03**	0.7256	0.7257	0.01	3.17E-01
S_{MAX}	0.8554	0.8554	0.01	3.93E-01	0.6714	0.6693	**-0.31**	**1.97E-06**	0.7258	0.7257	-0.01	4.06E-01
U_{AVG}	0.8062	0.8065	0.03	3.95E-01	0.6602	0.6609	**0.11**	**5.64E-08**	0.6687	0.6768	**1.21**	**1.10E-17**
Z_{AVG}	0.7257	0.7305	**0.67**	**7.13E-184**	0.6520	0.6521	0.02	4.04E-01	0.6884	0.6919	**0.51**	**3.82E-54**
W_{AVG}	0.7545	0.7814	**3.56**	**1.36E-07**	0.6500	0.6452	-0.74	3.17E-01	0.6712	0.6900	**2.79**	**2.83E-03**
N_{AVG}	0.7500	0.7546	**0.61**	**6.60E-206**	0.6505	0.6543	**0.58**	**1.43E-38**	0.6946	0.6985	**0.56**	**4.24E-78**
XN_{AVG}	0.7653	0.7700	**0.62**	**2.88E-152**	0.6485	0.6527	**0.65**	**1.58E-36**	0.7006	0.7041	**0.50**	**5.09E-74**
L_{AVG}	0.7435	0.7481	**0.62**	**9.95E-196**	0.6445	0.6486	**0.64**	**1.38E-36**	0.6776	0.6810	**0.50**	**1.66E-53**
XL_{AVG}	0.7577	0.7625	**0.62**	**4.89E-146**	0.6429	0.6476	**0.73**	**3.31E-32**	0.6854	0.6888	**0.49**	**7.08E-55**
S_{AVG}	0.7486	0.7495	**0.12**	**1.16E-13**	0.6498	0.6495	-0.05	3.38E-01	0.6808	0.6804	-0.05	2.41E-01
D_{DIC}	0.8381	0.8390	**0.12**	**2.52E-115**	0.6916	0.6926	**0.14**	**1.26E-17**	0.7776	0.7785	**0.10**	**2.53E-22**
Z_{DIC}	0.8323	0.8331	**0.11**	**5.67E-115**	0.6884	0.6885	0.02	1.47E-01	0.7802	0.7810	**0.10**	**2.48E-22**
W_{DIC}	0.8277	0.8282	**0.07**	**1.06E-33**	0.6802	0.6802	0.01	2.87E-01	0.7671	0.7673	**0.03**	**1.21E-03**
U_{DIC}	0.8311	0.8349	**0.45**	**3.98E-20**	0.6802	0.6804	0.02	1.50E-02	0.7589	0.7621	**0.42**	**1.69E-10**
D_{GIC}	0.8381	0.8390	**0.12**	**3.10E-154**	0.6916	0.6926	**0.14**	**9.39E-24**	0.7776	0.7785	**0.10**	**6.51E-30**
Z_{GIC}	0.8323	0.8331	**0.11**	**2.28E-165**	0.6884	0.6885	0.02	1.15E-01	0.7802	0.7810	**0.10**	**3.29E-30**
W_{GIC}	0.8277	0.8282	**0.07**	**1.07E-47**	0.6802	0.6802	0.01	2.39E-01	0.7671	0.7673	**0.03**	**2.90E-04**
U_{GIC}	0.8311	0.8349	**0.45**	**6.94E-22**	0.6802	0.6804	**0.02**	**4.07E-03**	0.7589	0.7621	**0.42**	**5.71E-12**
D_{UIC}	0.8346	0.8334	-0.14	3.69E-01	0.6930	0.6928	-0.03	4.05E-01	0.7757	0.7675	**-1.07**	**3.27E-04**
Z_{UIC}	0.8287	0.8272	-0.18	3.40E-01	0.6869	0.6870	0.02	4.05E-01	0.7783	0.7695	**-1.13**	**1.19E-04**
W_{UIC}	0.8239	0.8225	-0.17	3.40E-01	0.6770	0.6762	-0.12	4.05E-01	0.7634	0.7505	**-1.69**	**1.16E-06**
U_{UIC}	0.8286	0.8319	0.40	3.17E-02	0.6772	0.6760	-0.18	4.04E-01	0.7562	0.7526	-0.47	1.01E-01
R_{OR}	0.8246	0.8264	**0.21**	**2.16E-03**	0.6525	0.6644	**1.83**	**1.38E-09**	0.8062	0.8143	**1.01**	**4.11E-12**
I_{UR}	0.8260	0.8262	0.03	3.95E-01	0.6655	0.6649	-0.10	4.05E-01	0.8158	0.8248	**1.11**	**2.55E-12**

Table 6. Improvements of performances of *FS** measures over *FS* measures predicting disease genes on benchmark dataset of BP, MF, CC ontologies

Experiments	DiseaseGenes_BP				DiseaseGenes_MF				DiseaseGenes_CC			
Methods	*FS*	*FS**	improve-ment(%)	FDR p-value	*FS*	*FS**	improve-ment(%)	FDR p-value	*FS*	*FS**	improve-ment(%)	FDR p-value
U_{ABM}	0.7714	0.7732	**0.23**	**7.36E-09**	0.7081	0.7087	**0.08**	**5.64E-03**	0.6834	0.6823	**-0.17**	**1.88E-04**
Z_{ABM}	0.7514	0.7536	**0.30**	**6.59E-17**	0.6971	0.6982	**0.15**	**2.86E-04**	0.6698	0.6709	**0.16**	**5.15E-08**
W_{ABM}	0.7487	0.7589	1.37	7.37E-02	0.7023	0.6910	-1.60	5.23E-02	0.6818	0.6868	0.74	2.95E-01
N_{ABM}	0.7528	0.7552	**0.33**	**1.76E-24**	0.7036	0.7056	**0.29**	**2.31E-05**	0.6743	0.6761	**0.27**	**8.17E-20**
XN_{ABM}	0.7623	0.7649	**0.34**	**1.47E-19**	0.7034	0.7042	0.11	1.46E-01	0.6734	0.6742	**0.12**	**1.88E-04**
L_{ABM}	0.7409	0.7438	**0.39**	**5.68E-31**	0.6944	0.6964	**0.29**	**4.92E-04**	0.6688	0.6703	**0.22**	**4.05E-14**
XL_{ABM}	0.7495	0.7530	**0.46**	**1.32E-30**	0.6975	0.6983	0.12	1.94E-01	0.6689	0.6698	**0.13**	**6.84E-05**
S_{ABM}	0.7420	0.7437	**0.23**	**1.47E-24**	0.6986	0.6981	-0.08	3.78E-01	0.6711	0.6708	-0.04	2.53E-01
U_{BMA}	0.7942	0.7954	**0.15**	**1.20E-04**	0.7357	0.7357	0.00	4.06E-01	0.7032	0.7029	-0.04	3.17E-01
Z_{BMA}	0.7983	0.7989	0.07	3.68E-02	0.7292	0.7302	**0.13**	**2.10E-03**	0.6923	0.6929	**0.09**	**2.55E-03**
W_{BMA}	0.7874	0.7769	-1.34	4.37E-02	0.7325	0.7122	**-2.77**	**1.40E-04**	0.7006	0.6978	-0.39	3.91E-01
N_{BMA}	0.8008	0.8019	**0.13**	**1.26E-06**	0.7330	0.7344	**0.20**	**1.60E-03**	0.6968	0.6979	**0.16**	**1.12E-08**
XN_{BMA}	0.8058	**0.8065**	0.09	1.25E-02	0.7282	0.7284	0.04	3.95E-01	0.6919	0.6924	0.07	2.58E-02
L_{BMA}	0.7922	0.7937	**0.19**	**1.16E-11**	0.7265	0.7277	0.17	3.52E-02	0.6914	0.6932	**0.26**	**3.74E-22**
XL_{BMA}	0.8005	0.8015	**0.13**	**2.81E-04**	0.7228	0.7236	0.10	2.04E-01	0.6881	0.6891	**0.14**	**2.94E-06**
S_{BMA}	0.7923	0.7934	**0.14**	**1.74E-10**	0.7303	0.7290	-0.18	8.56E-02	0.6935	0.6933	-0.03	3.17E-01
U_{MAX}	0.7584	0.7599	**0.19**	**3.30E-04**	0.5926	0.5925	-0.01	4.06E-01	0.6059	0.6059	0.00	4.06E-01
Z_{MAX}	0.7634	0.7638	0.04	1.65E-01	0.5940	0.5939	-0.01	4.06E-01	0.6040	0.6040	0.00	4.06E-01
W_{MAX}	0.7598	0.7468	-1.71	1.39E-02	0.5936	0.5882	-0.91	2.73E-01	0.6008	0.6076	1.12	1.58E-01
N_{MAX}	0.7665	0.7668	0.03	2.67E-01	0.5910	0.5910	0.01	4.06E-01	0.6034	0.6033	-0.01	4.06E-01
XN_{MAX}	0.7652	0.7658	0.08	1.29E-02	0.5918	0.5917	-0.02	4.06E-01	0.6023	0.6023	0.00	4.06E-01
L_{MAX}	0.7675	0.7678	0.05	1.14E-01	0.5916	0.5916	0.01	4.06E-01	0.6041	0.6041	0.00	4.06E-01
XL_{MAX}	0.7632	0.7638	0.08	3.68E-02	0.5921	0.5920	-0.02	4.06E-01	0.6025	0.6023	-0.04	2.53E-01
S_{MAX}	0.7683	0.7679	-0.04	8.39E-02	0.5918	0.5917	-0.01	4.06E-01	0.6040	0.6040	0.01	4.06E-01
U_{AVG}	0.7564	0.7599	**0.46**	**1.03E-27**	0.6795	0.6815	**0.29**	**2.45E-18**	0.6769	0.6783	0.21	1.03E-02
Z_{AVG}	0.7247	0.7287	**0.56**	**1.01E-33**	0.6766	0.6791	**0.37**	**1.48E-12**	0.6541	0.6556	**0.24**	**9.82E-14**
W_{AVG}	0.7147	0.6674	**-6.62**	**4.30E-14**	0.6665	0.6358	**-4.62**	**2.03E-06**	0.6797	0.6581	**-3.17**	**2.56E-03**
N_{AVG}	0.7270	0.7308	**0.51**	**2.30E-34**	0.6840	0.6854	0.21	9.55E-02	0.6562	0.6580	**0.28**	**1.26E-16**
XN_{AVG}	0.7293	0.7334	**0.57**	**7.45E-32**	0.6838	0.6841	0.05	4.06E-01	0.6602	0.6619	**0.25**	**5.56E-12**
L_{AVG}	0.7173	0.7214	**0.57**	**2.47E-39**	0.6798	0.6808	0.15	2.61E-01	0.6514	0.6530	**0.24**	**4.67E-14**
XL_{AVG}	0.7186	0.7233	**0.66**	**1.79E-39**	0.6791	0.6789	-0.03	4.06E-01	0.6543	0.6566	**0.35**	**3.74E-22**
S_{AVG}	0.7207	0.7218	**0.15**	**1.79E-06**	0.6836	0.6826	-0.14	3.63E-01	0.6528	0.6532	0.06	1.85E-01
D_{DIC}	0.7695	0.7707	**0.16**	**5.26E-26**	0.7224	0.7238	**0.20**	**1.58E-10**	0.6770	0.6783	**0.20**	**1.04E-11**
Z_{DIC}	0.7707	0.7718	**0.15**	**1.42E-25**	0.7055	0.7060	**0.07**	**1.95E-03**	0.6761	0.6770	**0.12**	**6.84E-05**
W_{DIC}	0.7477	0.7478	0.02	2.93E-01	0.6998	0.6999	0.03	2.13E-01	0.6654	0.6658	0.06	2.35E-02
U_{DIC}	0.7395	0.7434	**0.52**	**4.86E-11**	0.7032	0.7037	**0.06**	**8.95E-08**	0.6768	0.6720	**-0.71**	**2.52E-18**
D_{GIC}	0.7695	0.7707	**0.16**	**1.68E-29**	0.7224	0.7238	**0.20**	**4.70E-12**	0.6770	0.6783	**0.20**	**1.09E-13**
Z_{GIC}	0.7707	0.7719	**0.15**	**1.69E-32**	0.7055	0.7060	**0.07**	**6.07E-04**	0.6761	0.6770	**0.12**	**1.62E-05**
W_{GIC}	0.7477	0.7478	0.02	2.67E-01	0.6998	0.7000	0.03	1.84E-01	0.6654	0.6658	0.06	1.67E-02
U_{GIC}	0.7395	0.7434	**0.52**	**1.90E-12**	0.7032	0.7037	**0.06**	**1.40E-09**	0.6768	0.6720	**-0.71**	**8.54E-22**
D_{UIC}	0.7519	0.7551	0.43	2.39E-01	0.7127	0.7237	**1.54**	**1.60E-03**	0.6654	0.6764	**1.65**	**3.33E-03**
Z_{UIC}	0.7536	0.7549	0.17	3.87E-01	0.6995	0.7032	0.53	2.61E-01	0.6650	0.6756	**1.60**	**5.04E-03**
W_{UIC}	0.7291	0.7300	0.12	3.99E-01	0.6931	0.6957	0.39	3.56E-01	0.6531	0.6630	**1.52**	**8.92E-03**
U_{UIC}	0.7286	0.7289	0.04	3.99E-01	0.6983	0.7042	0.85	4.77E-02	0.6700	0.6743	0.65	2.02E-01
R_{OR}	0.8062	0.7975	-1.08	1.29E-01	0.7229	**0.7541**	**4.31**	**1.30E-07**	0.7031	**0.7064**	0.46	3.91E-01
I_{UR}	0.8030	0.7737	**-3.66**	**3.35E-15**	0.7202	0.7290	1.22	1.04E-01	0.6988	0.6914	-1.06	1.72E-01

Supplementary Figures

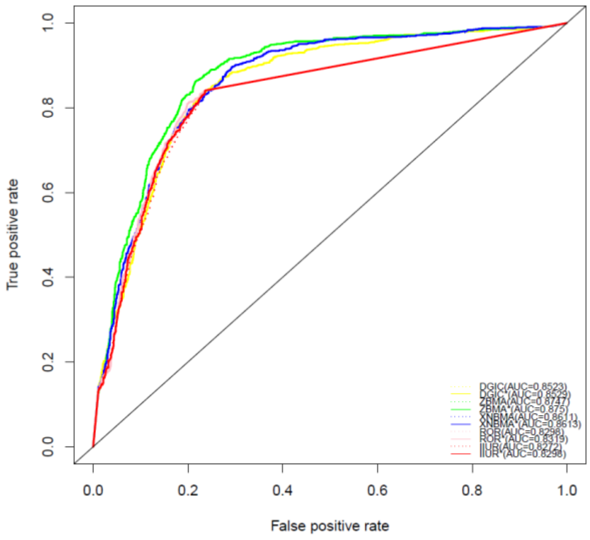

Figure 1.1 ROC curves and AUC values indicating performance of predicting protein interactions of human PPI dataset by *FS* and *FS** measures on BP ontology

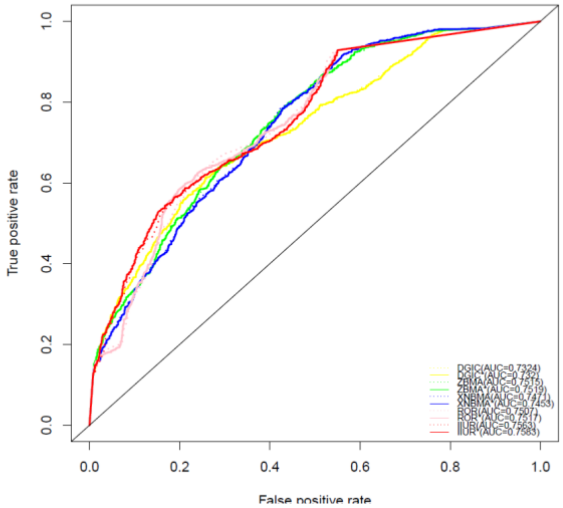

Figure 1.2 ROC curves and AUC values indicating performance of predicting protein interactions of human PPI dataset by *FS* and *FS** measures on MF ontology

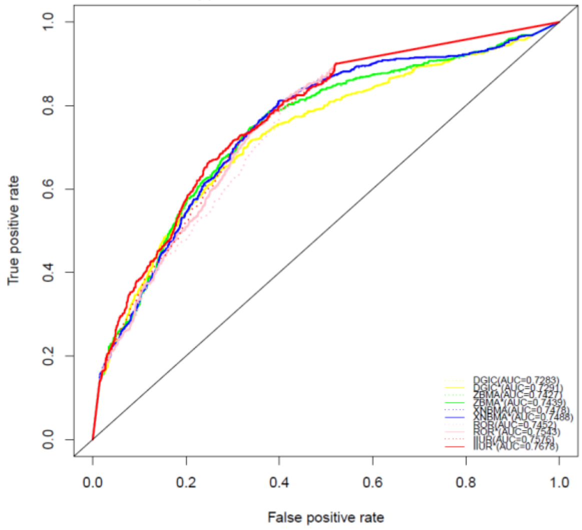

Figure 1.3 ROC curves and AUC values indicating performance of predicting protein interactions of human PPI dataset by *FS* and *FS** measures on CC ontology

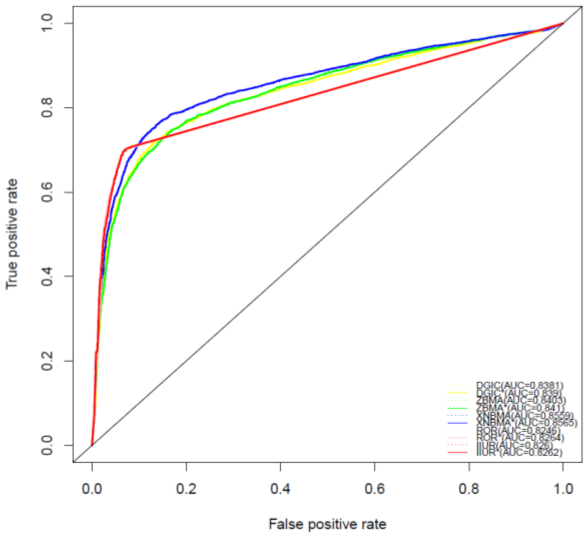

Figure 2.1 ROC curves and AUC values indicating performance of predicting protein interactions of yeast PPI dataset by *FS* and *FS** measures on BP ontology

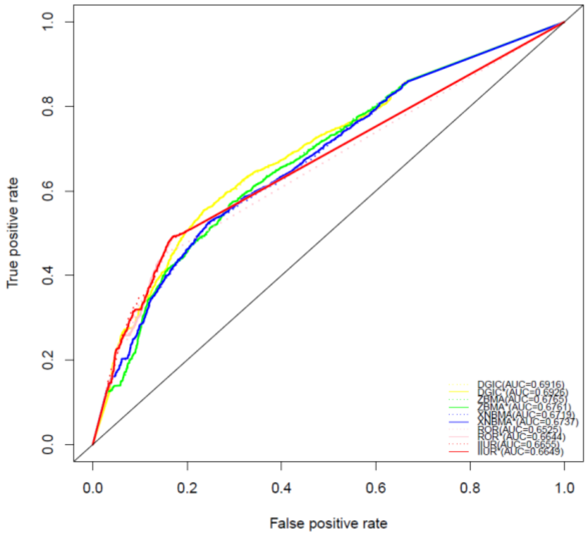

Figure 2.2 ROC curves and AUC values indicating performance of predicting protein interactions of yeast PPI dataset by *FS* and *FS** measures on MF ontology

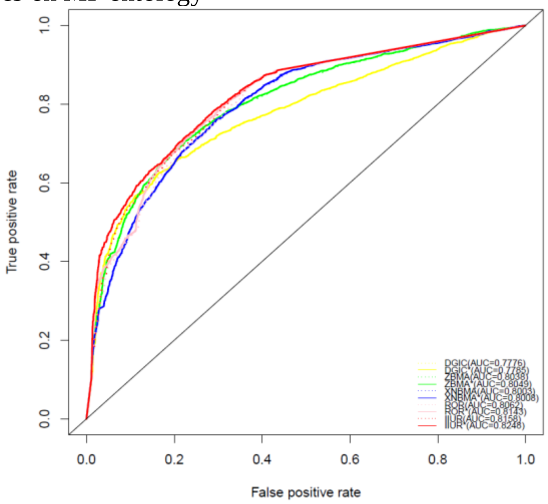

Figure 2.3 ROC curves and AUC values indicating performance of predicting protein interactions of yeast PPI dataset by *FS* and *FS** measures on CC ontology

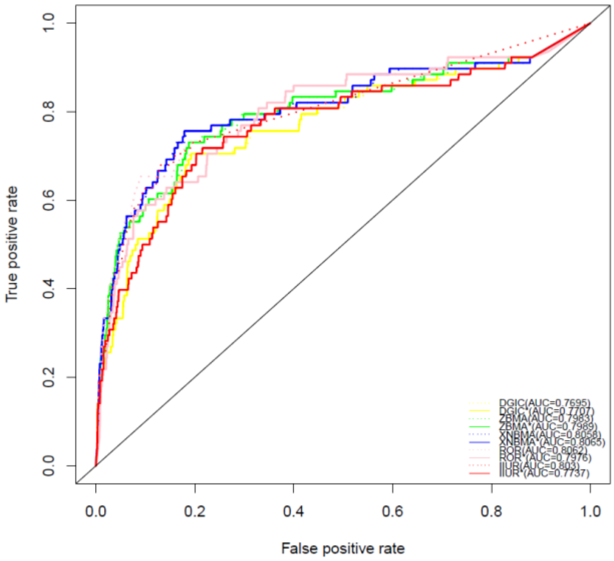

Figure 3.1. ROC curves and AUC values indicating performance of predicting disease genes on benchmark dataset by *FS* and *FS** measures on BP ontology

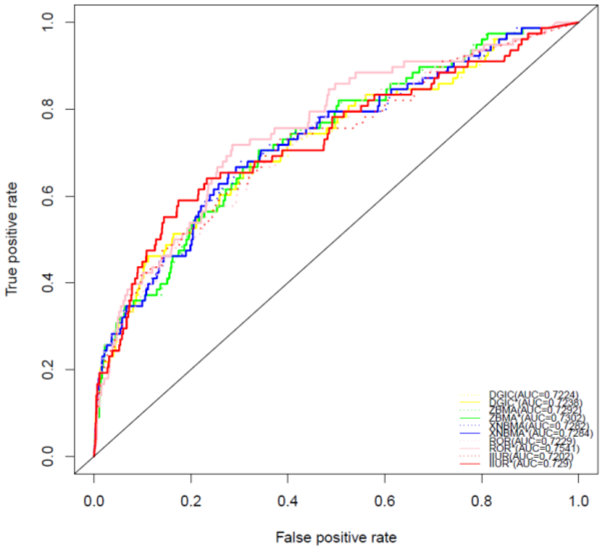

Figure 3.2. AUC values indicating performance of predicting disease genes on benchmark dataset by *FS* and *FS** measures on MF ontology

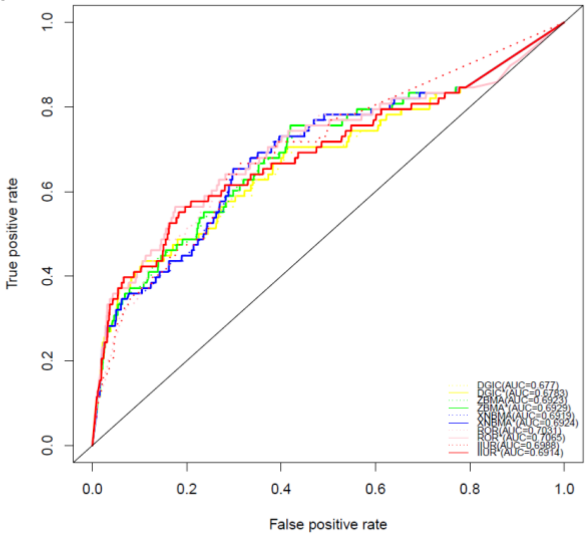

Figure 3.3. AUC values indicating performance of predicting disease genes on benchmark dataset by *FS* and *FS** measures on CC ontology
